# Deformation Prediction of 4D‐Printed Active Composite Structures Based on Data Mining

**DOI:** 10.1002/advs.202516989

**Published:** 2025-11-26

**Authors:** Mengtao Wang, Yifan Xu, Zaiyang Liu, Hidemitsu Furukawa, Zhongkui Wang, Ren Xu, Lin Meng

**Affiliations:** ^1^ Department of Electronic and Computer Engineering Ritsumeikan University Shiga 525‐8577 Japan; ^2^ Department of Robotics Ritsumeikan University Shiga 525‐8577 Japan; ^3^ Department of Mechanical Systems Engineering Yamagata University Yonezawa 992‐8510 Japan; ^4^ School of Medicine Xiamen University Xiamen Fujian 361100 China

**Keywords:** 4D printing, active composites, data mining, deformation prediction, voxel assembly

## Abstract

Voxelizing active composite structures and controlling voxel‐level material properties via 4D printing significantly expand design possibilities. However, as the number of voxels increases, the design space grows exponentially, posing significant challenges for predicting structural deformation. Here, a scalable deformation prediction method based on data mining is proposed. This method constructs a feature database using manually extracted features and employs the proposed curvature‐driven sequence point generation (CSPG) algorithm to predict deformations for voxel encodings of arbitrary length. Compared with the traditional finite element (FE) method, this approach significantly improves prediction efficiency, completing a single task within a second. In contrast to deep learning (DL) methods, this method improves prediction accuracy and effectively addresses the limited generalization ability of DL models when applied to structures beyond the training domain. To enhance usability, an interactive web‐based platform is developed that allows users to customize voxel encodings and obtain end‐to‐end predictions. In addition to serving as an efficient tool for deformation prediction of active composite structures, this work introduces a novel pathway for the optimal design of complex intelligent structures.

## Graphical abstract

A curvature‐driven sequence point generation (CSPG) algorithm based on data mining is proposed to predict the deformation of double‐layer voxelized composite structures of arbitrary lengths. In addition, the CSPG algorithm is applied to predict the deformation of 2D and 3D structures assembled from beam elements, and its effectiveness is validated through 4D‐printed hydrogel experiments. This study provides an efficient tool for deformation prediction of active composite structures.

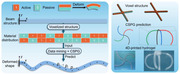

## 1 Introduction

**Figure 1:**
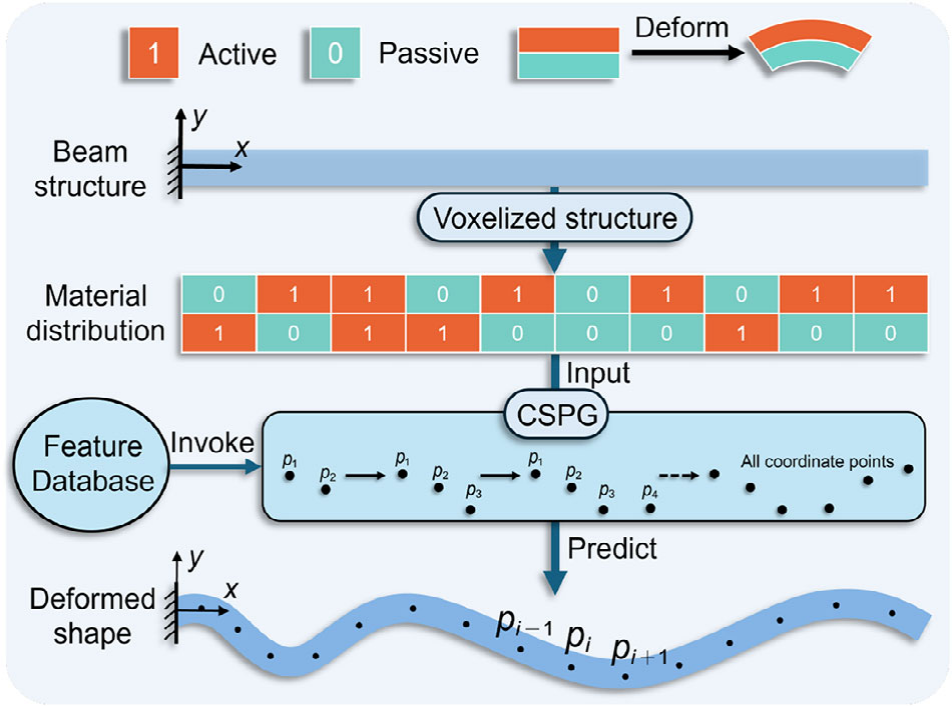
Schematic illustration of deformation prediction for voxelized composite structures based on the CSPG algorithm. The bilayer structure, composed of active material (encoded as “1”) and passive material (encoded as “0”), deforms under external stimuli due to differences in expansion rates. A beam structure of arbitrary length is voxelized and encoded before being input into the CSPG algorithm. The algorithm retrieves geometric features from a pre‐constructed feature database to iteratively generate all deformation coordinates, thereby enabling efficient prediction of the deformation trajectory.

Building upon 3D printing, 4D printing introduces the dimension of time, enabling printed structures to evolve dynamically in response to environmental changes. The technology relies on a class of stimuli‐responsive materials, such as shape memory polymers (SMPs),^[^ ^1^, ^2^, ^3^ ^]^ liquid crystal elastomers (LCEs),^[^ ^4^, ^5^, ^6^ ^]^ and hydrogels,^[^ ^7^, ^8^, ^9^ ^]^ to drive structural transformations in shape or function under stimulus conditions, such as temperature,^[^ ^10^, ^11^ ^]^ light,^[^ ^12^, ^13^ ^]^ humidity,^[^ ^14^, ^15^ ^]^ or magnetic fields,^[^ ^16^, ^17^ ^]^ thus significantly enhancing the dynamic adaptability of the materials. Traditional continuous materials exhibit limitations in achieving localized and differential responses. In contrast, voxelized design discretizes structures into individually controllable material units, allowing the fine‐tuning of the properties of each voxel through 4D printing techniques. This enables globally programmable deformation and functional behaviors, thereby offering enhanced flexibility and precision in structural and functional design.^[^ ^18^, ^19^, ^20^ ^]^ Taking the structure illustrated in **Figure** **1** as an example, the voxelized bilayer structure is composed of active and passive materials with differing expansion ratios, which undergo deformation under external environmental stimuli. By further discretizing a beam‐shaped structure into an assembly of voxel units and randomly assigning material properties to each unit, a spatially distributed system with high structural complexity can be constructed. As the number of voxels increases, the design space expands exponentially, providing substantial freedom for material configuration. However, this also introduces a significant challenge in accurately predicting the deformation behavior of the structure.

With the rapid development of high‐performance computing technology, numerical approximation methods have become the core approach to simulate real physical systems, providing an effective way for modeling and predicting the mechanical behavior of complex structures. Among these methods, finite element (FE) analysis stands out as one of the most widely adopted techniques.^[^ ^21^ ^]^ It has been extensively developed in various fields, including structural mechanics,^[^ ^22^, ^23^ ^]^ heat transfer,^[^ ^24^, ^25^ ^]^ biomedical engineering,^[^ ^26^, ^27^ ^]^ materials science,^[^ ^28^, ^29^ ^]^ demonstrating strong adaptability in multi‐physics modeling and response analysis. In predicting the mechanical response of active composites, FE methods also play a crucial role in effectively characterizing the deformation behavior of complex structures under environmental stimuli, thus significantly reducing experimental costs and shortening the design cycle.^[^ ^30^, ^31^, ^32^ ^]^ However, when dealing with structurally complex systems or large‐scale predictive tasks, the high computational cost and low efficiency of traditional numerical methods become major bottlenecks, thereby hindering their application in rapid prediction and high‐throughput structural design. For example, Hamel et al.^[^ ^33^ ^]^ integrated FE analysis with evolutionary algorithms for the target shape design of active composite materials. Although parallel computing strategies were adopted to enhance efficiency, the overall simulation process remained highly time‐consuming due to the iterative evaluation of a large number of candidate structures. Similarly, Athinarayanarao et al.^[^ ^34^ ^]^ developed an inverse design framework by combining FE methods with evolutionary algorithms to solve inverse design problems in voxelized structures with voids. However, the simulation process remained computationally intensive.

In recent years, the rapid advancement of deep learning (DL) technologies has opened new avenues for constructing efficient predictive models of structural behavior.^[^ ^35^, ^36^ ^]^ Unlike traditional numerical methods that rely on explicit physical models and analytical formulations, DL can autonomously learn the underlying relationships between structural design parameters and performance responses from large volumes of sample data, without the need to construct explicit physical models. This capability enables fast and accurate prediction of complex system behaviors.^[^ ^37^ ^]^ As a representative data‐driven approach, DL has demonstrated strong applicability in the field of composite materials. It has been widely employed in tasks such as mechanical property prediction,^[^ ^38^, ^39^, ^40^ ^]^ stress/strain field prediction,^[^ ^41^, ^42^, ^43^, ^44^ ^]^ and deformation prediction.^[^ ^45^, ^46^ ^]^ These applications have enabled significant progress in addressing the limitations of traditional methods, particularly with respect to modeling complexity and computational cost.

For voxelized active composite structures, existing studies have shown that DL‐based prediction methods can significantly enhance computational efficiency while maintaining high accuracy. For example, Sun et al.^[^ ^47^ ^]^ integrated DL with evolutionary algorithms to enable both forward deformation prediction and inverse design of 4D‐printed active composite beams. In their framework, the forward prediction module employed a recurrent neural network (RNN) model, which achieved high accuracy across various structural configurations (24 × 4, 40 × 2, 40 × 4), thereby significantly enhancing the efficiency of the inverse design process. Tang et al.^[^ ^48^ ^]^ introduced generative adversarial networks (GANs) for predicting the deformation and stress fields of programmable active composite materials. Their approach demonstrated strong predictive capability and efficiency advantages for 20 × 20 voxelized structures. In addition, Sun et al.^[^ ^49^ ^]^ proposed a DL model based on residual networks combined with evolutionary algorithms to enable efficient inverse design of active composite plates with dimensions 15 × 15 × 2. In our previous work,^[^ ^50^ ^]^ we proposed a rapid inverse design method that integrates convolutional neural networks (CNNs) with a progressive evolutionary strategy. Applied to composite beam structures with dimensions of 10 × 2, this proposed approach achieved significant improvements in both efficiency and accuracy compared to traditional evolutionary algorithms.

The above studies demonstrate the significant potential of DL in replacing the FE method for structural forward prediction, particularly in enhancing simulation efficiency and accelerating the design process.^[^ ^51^ ^]^ However, these methods generally face a common limitation: their predictive capability is constrained by the structural dimensions present in the training data. Since DL models inherently rely on learning the mapping from inputs to outputs based on fixed‐size training samples, their ability to generalize across varying structural dimensions remains limited. Most existing models struggle to generalize to unseen structural configurations, particularly when encountering longer sequential structures that exceed the training scale, often resulting in a substantial decline in prediction accuracy. This limitation stems from the fact that DL methods primarily rely on memorizing positional features of the input, rather than capturing the underlying physical principles that govern the relationship between encoded structures and global deformation behavior. As a result, when the structural length exceeds the range covered by the training dataset, the model struggles to perform principle‐based extrapolation, thereby limiting its applicability to scalable and cross‐scale structural modeling tasks.

To address the scalability limitations of DL methods in modeling 4D‐printed active composite structures, this study proposes a curvature‐driven sequence point generation (CSPG) algorithm based on data mining. Unlike DL models that rely on learning from data distributions, the CSPG algorithm explicitly extracts key geometric features from simulation data that characterize structural deformation, and leverages these features to sequentially generate deformation coordinate points. Specifically, the CSPG algorithm begins by extracting features from FE simulation data to construct a feature database that captures the deformation curvatures and adjacent point distances associated with various voxel encoding patterns. By specifying initial conditions and employing a sequential recommendation strategy, the algorithm calculates the position of each subsequent coordinate point based on the geometric location and feature values of the preceding two points. In this manner, it progressively generates the complete deformation coordinates set for structures of arbitrary length, as illustrated in Figure 1. Compared to traditional FE methods, the CSPG algorithm eliminates the need to construct complete physical field models or perform complex numerical iterations. Instead, it leverages geometric feature information to enable rapid inference, significantly improving prediction efficiency, with each prediction completed within 1 second. Furthermore, the CSPG algorithm achieves prediction accuracy comparable to DL models on short structures, while significantly outperforming them on longer structures. More importantly, it enables deformation prediction for structures of arbitrary length without requiring additional training data, model retraining, or network reconfiguration. To enhance the practicality and operational convenience of this prediction method, we further developed an interactive web‐based prediction platform. This platform enables users to customize voxel encodings and assembly schemes, facilitating end‐to‐end deformation prediction of the structures. This work not only provides an efficient tool for deformation prediction of active composite structures but also offers a novel approach for the optimal design of complex intelligent systems.

## 2 Results

### 2.1 Dataset Generation and Manual Feature Extraction

**Figure 2:**
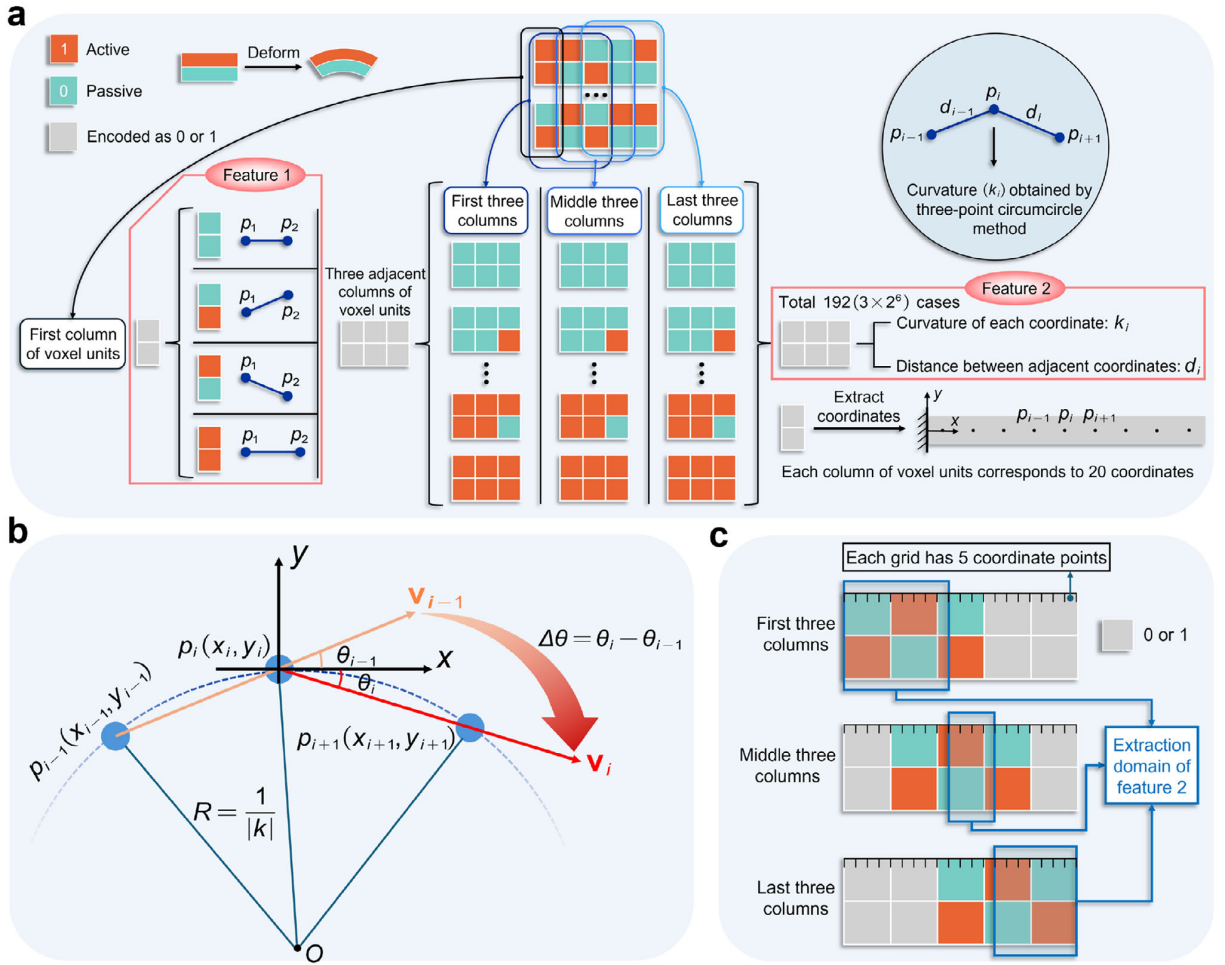
Schematic illustration of manual feature extraction and feature database construction. a) A total of 640 voxelized structures with a size of 2 × 5 are randomly generated, and their deformation coordinate points are obtained by FE simulation. The voxel encodings are divided into four types: the first column, the first three columns, the middle three columns, and the last three columns. Two types of geometric features are extracted from each segment: Feature 1 refers to the initial two coordinate points within the segment; Feature 2 includes the curvature of each point and the distance between adjacent points. b) Method for curvature calculation. The absolute value of the curvature of point *p*_*i*_ is determined using the three‐point circumcircle method, while the sign of the curvature is determined based on the angle Δθ between adjacent vectors $v_{i-1}$ and $v_{i}$. c) Strategy for selecting feature values. Feature 2 is extracted from the region marked in the figure to effectively avoid curvature errors caused by non‐smooth transitions in the deformation profile.

In this study, we design a voxelized bilayer active composite structure composed of two materials with different properties. As illustrated in **Figure** **2**, the active material (encoded as “1”) exhibits a higher expansion ratio, whereas the passive material (encoded as “0”) exhibits a lower one. Under external environmental stimuli, the mismatch in expansion ratios between the two materials induces a bending deformation of the active layer toward the passive side. By randomly distributing these two materials within a voxel structure of size 2 × *N* (*N* denotes the number of columns), a composite structure with a complex spatial distribution can be formed. For any voxel sequence of length *N*, there exist 2^2*N*^ possible material combinations. As *N* increases, the design space expands exponentially. This vast design space provides a high degree of design freedom, while simultaneously posing significant challenges for accurate deformation prediction.

To address the problem of deformation prediction for structures with arbitrary length, we propose a CSPG algorithm. This method manually extracts two categories of critical features: (1) the initial two coordinate points after deformation, denoted as *p*_1_ and *p*_2_; (2) the curvature values *k*_*i*_ at each coordinate point and the distances *d*_*i*_ between adjacent points. To obtain these features, we perform FE simulations of various voxel‐encoded structures to generate deformation data. For each column in the voxelized structure, 20 coordinate points are uniformly sampled, forming a dataset in which the voxel encodings serve as inputs and the corresponding deformed coordinates as outputs. During FE simulations, when only a single column of voxels is considered (*N* = 1), there are four possible encoding combinations. In this case, each encoding yields a fixed set of *k*_*i*_ and *d*_*i*_. Similarly, when *N* = 3, there are 64 possible encoding combinations, each of which follows the same deformation law. To facilitate modeling and analysis of voxel encoding sequences of arbitrary length, we divide them locally based on three adjacent columns of voxel units and generalize these into three distinct cases:

1. The first three columns: Located at the beginning of the sequence, their deformation is influenced only by the encodings on the right.
2. The last three columns: Located at the end of the sequence, their deformation is influenced only by the encodings on the left.
3. The middle three columns: All remaining neighboring three‐column combinations within the structure, their deformation is influenced by both left and right neighboring encodings.

This segmentation strategy is designed to consider the distinct deformation behaviors exhibited by substructures at different positions under varying boundary conditions, thereby providing a structured foundation for subsequent modeling and prediction. As shown in Figure 2a, we randomly generated 640 voxel‐coded samples of size 2 × 5 and simulated their deformation using the FE method. The deformed shape consists of a sequence of surface sampling points *p*_*i*_ = (*x*_*i*_, *y*_*i*_). By extracting the first two deformed coordinate points *p*_1_ and *p*_2_ for each sample, we observed that once the encoding of the first column is determined, the positions of these initial two points are also fixed. The results are presented in Figure S1 (Supporting Information). Since each column has four possible encoding combinations, there are only four types of feature 1. Next, each 2 × 5 voxel structure is divided into three categories based on the previously defined segmentation: first three columns, middle three columns, and last three columns. Each category contains 64 encoding combinations, resulting in a total of 192 distinct cases. For each case, we extract all deformed coordinate points and compute their corresponding curvature values *k*_*i*_ and adjacent distance *d*_*i*_. The adjacent distances *d*_*i*_ are calculated according to Equation (1),

(1) $$\begin{array}{c} d_{i}=\sqrt{{\left(x_{i+1}-x_{i}\right)}^{2}+{\left(y_{i+1}-y_{i}\right)}^{2}}. \end{array}$$

The computation of the curvature value *k*_*i*_ is illustrated in Figure 2b. The figure shows three adjacent deformation coordinate points {*p*_*i* − 1_, *p*_*i*_, *p*_*i* + 1_}, which can determine a unique circumcircle. Based on the three‐point circumcircle method, the absolute curvature value |*k*_*i*_| at point *p*_*i*_ can be computed using Equation (2), where $S_{\Delta p_{i-1}p_{i}p_{i+1}}$ denotes the area of the triangle formed by points *p*_*i* − 1_, *p*_*i*_ and *p*_*i* + 1_, and *d*(*p*_*j*_, *p*_*k*_) represents the Euclidean distance between *p*_*j*_ and *p*_*k*_. To further determine the sign of the curvature, a local 2D coordinate system is established with the point *p*_*i*_ as the origin. In this coordinate system, two vectors $v_{i-1}$ and $v_{i}$ are constructed using Equation (3). Then, the directional angles θ_*i* − 1_ and θ_*i*_ of these two vectors are computed using the four‐quadrant arctangent function, as given in Equation (4). The angle between the two vectors is denoted as $\Delta \theta =\theta_{i}-\theta_{i-1}$, and the curvature sign *k*_*sign*_ is determined using Equation (5). Finally, the curvature value *k*_*i*_ at point *p*_*i*_ is determined using Equation (6),

(2) $$\begin{array}{c} \left|k_{i}\right|=\frac{4S_{\Delta p_{i-1}p_{i}p_{i+1}}}{d\left(p_{i-1},p_{i}\right)d\left(p_{i},p_{i+1}\right)d\left(p_{i-1},p_{i+1}\right)} \end{array},$$

(3) $$\begin{array}{c} v_{i}=\left(x_{i+1}-x_{i},y_{i+1}-y_{i}\right)=\left(v_{i}^{\left(x\right)},v_{i}^{\left(y\right)}\right), \end{array}$$

(4) $$\begin{array}{c} \theta_{i}=\left\{\begin{array}{cc} \arctan\left(\frac{v_{i}^{\left(y\right)}}{v_{i}^{\left(x\right)}}\right) & \text{if}\;v_{i}^{\left(x\right)}>0\\ \arctan\left(\frac{v_{i}^{\left(y\right)}}{v_{i}^{\left(x\right)}}\right)+\pi & \text{if}\;v_{i}^{\left(x\right)}<0,\;v_{i}^{\left(y\right)}\geq 0\\ \arctan\left(\frac{v_{i}^{\left(y\right)}}{v_{i}^{\left(x\right)}}\right)-\pi & \text{if}\;v_{i}^{\left(x\right)}<0,\;v_{i}^{\left(y\right)}<0\\ +\frac{\pi }{2} & \text{if}\;v_{i}^{\left(x\right)}=0,\;v_{i}^{\left(y\right)}>0\\ -\frac{\pi }{2} & \text{if}\;v_{i}^{\left(x\right)}=0,\;v_{i}^{\left(y\right)}<0\\ \text{undefined} & \text{if}\;v_{i}^{\left(x\right)}=0,\;v_{i}^{\left(y\right)}=0 \end{array}\right.\;, \end{array}$$

(5) $$\begin{array}{c} k_{\text{sign}}=\left\{\begin{array}{cc} 1 & \text{if}\;\Delta \theta >0\\ -1 & \text{if}\;\Delta \theta <0 \end{array}\right.\;, \end{array}$$

(6) $$\begin{array}{c} k_{i}=k_{sign}\cdot \left|k_{i}\right| \end{array}.$$

**Figure 3:**
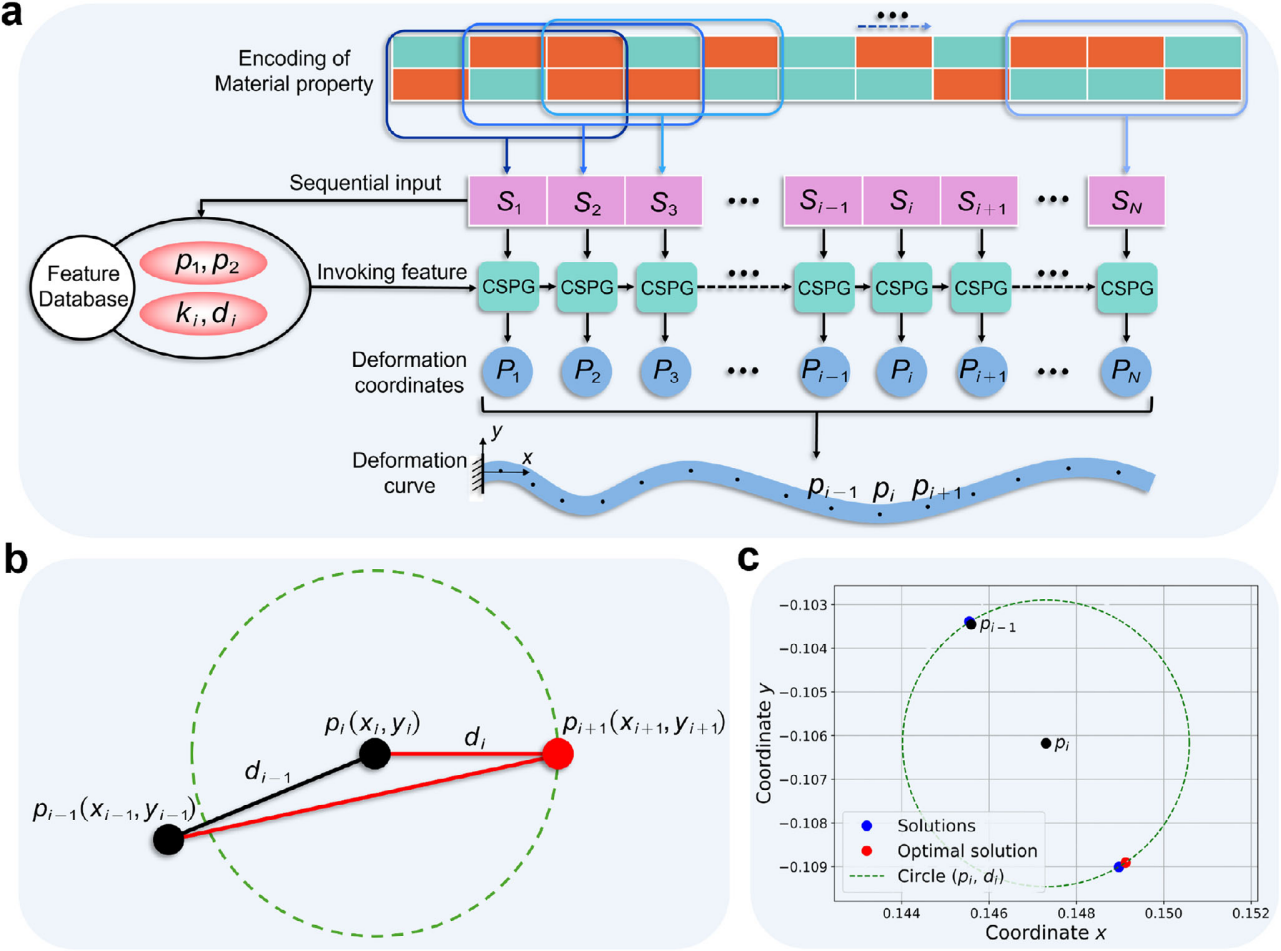
Illustration of deformation prediction using the CSPG algorithm. a) The voxel encoding is divided into a series of spatially ordered subsequences S, each composed of three adjacent columns. These subsequences are sequentially input into the feature database to invoke corresponding geometric features. The CSPG algorithm then incrementally generates all deformation coordinate points, ultimately forming the complete deformation trajectory. b) Schematic of the CSPG algorithm. Given the positions of the previous two points *p*_*i* − 1_ and *p*_*i*_, the distances *d*_*i* − 1_ and *d*_*i*_ between adjacent points, and the curvature of *p*_*i*_, the next coordinate point *p*_*i* + 1_ can be derived using Equation (7) and Equation (8). c) Practical solution example of the CSPG algorithm. The black points represent the known coordinates *p*_*i* − 1_ and *p*_*i*_. Based on Equation (7), four candidate positions for *p*_*i* + 1_ are computed. After further screening through Equation (8), the blue coordinate points are the invalid solutions that are excluded, and the red coordinate points are the finalized optimal solutions.

Since the CSPG algorithm employs a piecewise concatenation strategy, the extraction process of feature 2 must be segmented accordingly. As shown in Figure 2c, each column of the voxel encoding is uniformly divided into four grid regions, with each region containing five sampling points. Depending on the encoding location type, the feature extraction ranges are defined as follows:

1. The first three columns: Feature values are extracted from the first nine grid regions.
2. The middle three columns: Feature values are extracted from the four grid regions labeled in the figure.
3. The last three columns: Feature values are extracted from the last seven grid regions.

Through the segmented extraction operations described above, continuous local features are obtained and ultimately concatenated into a complete feature representation corresponding to a continuous five‐column encoding. It is important to note that the feature extraction segments are not aligned with entire columns but are instead staggered by one mesh region between each segment. This is because when the encodings of adjacent columns differ, the connecting region experiences inconsistent stress directions due to variations in material properties, leading to a noticeable extrusion effect at the boundary. This region, characterized by significant stress variation, is defined as the transition region. Deformation features within this region often exhibit non‐smooth behavior. Directly stitching features within this region would introduce significant errors. Therefore, by shifting one grid cell backward and performing stitching in a smoother region, errors can be reduced and the overall stability and accuracy of the prediction can be improved. The details of feature 2 (*k*_*i*_, *d*_*i*_) extraction are presented in Figures S2 and S3 (Supporting Information).

### 2.2 Curvature‐Driven Sequence Point Generation (CSPG) Algorithm

The extracted Feature 1 (*p*_1_ and *p*_2_) and Feature 2 (*k*_*i*_ and *d*_*i*_) are integrated to construct a feature database. This database is then utilized in conjunction with the CSPG algorithm to enable deformation prediction for voxel structures of arbitrary length. The overall prediction workflow is illustrated in **Figure** **3**. Specifically, Figure 3b demonstrates the principle of the CSPG algorithm: given two consecutive coordinate points *p*_*i* − 1_ and *p*_*i*_, and the known distance *d*_*i*_ between *p*_*i*_ and *p*_*i* + 1_, a circular arc centered at *p*_*i*_ with radius *d*_*i*_ can be drawn. The point *p*_*i* + 1_ must lie on the circumference of this arc. Furthermore, if the curvature *k*_*i*_ at *p*_*i*_ is also known, the exact location of *p*_*i* + 1_ can be uniquely determined using Equation (7).

(7) $$\begin{array}{c} \left\{\begin{array}{c} \left|k_{i}\right|=\frac{4S_{\Delta p_{i-1}p_{i}p_{i+1}}}{d_{i-1}\cdot d_{i}\cdot \sqrt{{\left(x_{i+1}-x_{i-1}\right)}^{2}+{\left(y_{i+1}-y_{i-1}\right)}^{2}}}\\ {\left(x_{i+1}-x_{i}\right)}^{2}+{\left(y_{i+1}-y_{i}\right)}^{2}=d_{i}^{2} \end{array}\right. \end{array}.$$

The CSPG algorithm is designed for voxel material encodings of arbitrary length, and its specific prediction process is illustrated in Figure 3a. The core idea is to divide the overall encoding into a series of sub‐sequences, each consisting of three adjacent columns in spatial order, denoted as $S=\left\{S_{1},\text{…},S_{i-1},S_{i},S_{i+1},\text{…},S_{N}\right\}$. Subsequently, these sub‐sequences are sequentially input into the constructed feature database to enable feature extraction and the generation of deformation coordinate points. At the beginning of the process, the first subsequence *S*_1_ is input into the feature database. Since the first column encoding of *S*_1_ is known, the initial two coordinate points *p*_1_ and *p*_2_ can be directly retrieved. Additionally, since *S*_1_ represents the first three‐column encoding, the corresponding curvature value *k*_*i*_ and the distance *d*_*i*_ between neighboring points can also be extracted. Based on this information, the CSPG algorithm sequentially generates the coordinates for the initial segment of the structure. Then, *S*_2_ ∼ *S*_*n* − 1_ are sequentially input into the feature database as the middle three‐column encodings. The corresponding *k*_*i*_ and *d*_*i*_ are extracted, and the CSPG algorithm is used to generate the deformation coordinate points for the middle segment. Finally, *S*_*n*_ is input into the feature database as the last three columns of encodings to complete the generation of end coordinate points. All predicted coordinate points are then sequentially concatenated to form the complete deformation trajectory curve.

It is important to emphasize that Equation (7) is a system of quadratic equations, which may yield up to four real solutions. Therefore, the optimal solution must be further identified to determine the corresponding coordinate point. To this end, the quadratic spline interpolation is performed for each candidate solution ${\hat{p}}_{i+1}\left({\hat{x}}_{i+1},{\hat{y}}_{i+1}\right)$ using the known points *p*_*i* − 1_ and *p*_*i*_, respectively. The estimated curvature ${\hat{k}}_{i}$ of the interpolated curves at *p*_*i*_ is calculated using Equation (8). Then, the estimated curvature ${\hat{k}}_{i}$ is compared with the corresponding actual curvature *k*_*i*_ from the feature database, and the error term $\left|k_{i}-{\hat{k}}_{i}\right|$ is used as an evaluation criterion. The smaller the error value, the more closely the candidate point matches the local geometric characteristics of the target deformation curve. Accordingly, the corresponding solution is selected as the current optimal solution.

(8) $$\begin{array}{c} {\hat{k}}_{i}=\frac{dx\cdot ddy-dy\cdot ddx}{{\left(dx^{2}+dy^{2}\right)}^{3/2}} \end{array}.$$

Figure 3c illustrates an example of the solution process using the CSPG algorithm. Given two known coordinate points, *p*_*i* − 1_ and *p*_*i*_, and the corresponding features (*k*_*i*_ and *d*_*i*_), an arc can be drawn with *p*_*i*_ as the center and *d*_*i*_ as the radius. The four candidate solutions obtained through Equation (7) lie on this arc, satisfying the geometric constraints. Then, interpolation is used to evaluate each candidate, and the one with the smallest error is selected as the next point *p*_*i* + 1_. This process is recursively repeated, using the two latest points as inputs, until the complete deformation curve is generated. Supplementary Movie 1 dynamically shows the solution process.

### 2.3 Prediction Performance of CSPG Algorithm

Unlike traditional models restricted to fixed structural lengths, the CSPG algorithm enables deformation prediction for structures of arbitrary length. To assess its performance under multi‐scale conditions, voxel encoding samples of five different lengths (2 × 5, 2 × 10, 2 × 15, 2 × 20, 2 × 30) were randomly generated, with 1000 samples per category. For each sample, the corresponding truth deformation trajectory was obtained through FE simulation. The predicted coordinate sequences (**x** ^*pred*^, **y** ^*pred*^) generated by the CSPG algorithm were then compared against these simulated truth sequences (**x** ^*true*^, **y** ^*true*^). Prediction accuracy was quantitatively evaluated using the mean squared error (MSE) between the predicted and simulated coordinate points, reflecting the deviation from the actual deformation curves.

(9) $$\begin{array}{c} MSE=\frac{1}{n}\sum_{i=1}^{n}\left[{\left(x_{i}^{true}-x_{i}^{pred}\right)}^{2}+{\left(y_{i}^{true}-y_{i}^{pred}\right)}^{2}\right] \end{array}.$$

**Figure 4:**
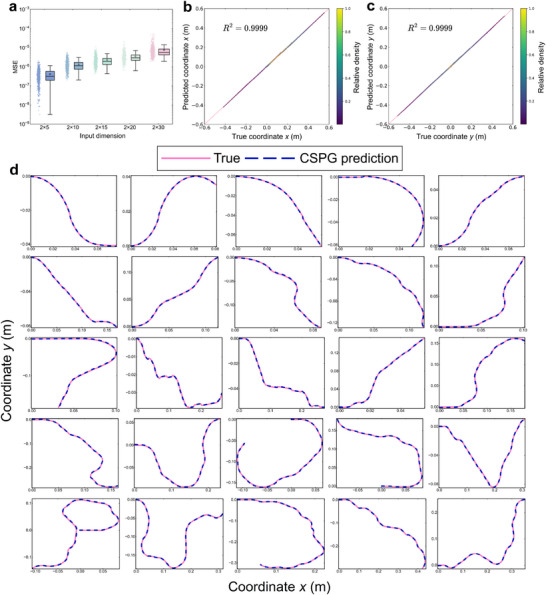
Deformation prediction performance of the CSPG algorithm. a) The CSPG algorithm was evaluated on voxel‐encoded structures of varying sizes, with 1000 randomly generated samples for each size. Across all samples, the predicted MSE consistently remained below 10^−5^, demonstrating excellent prediction accuracy. b,c) Scatter density plots of predicted coordinates and FE truth coordinates for 1000 samples of size 2 × 30. The coefficient of determination *R* ^2^ reaches 0.9999 for both the x and y coordinates. d) The prediction results for five randomly selected samples for each of the five voxel encoding sizes (2 × 5, 2 × 10, 2 × 15, 2 × 20, 2 × 30), corresponding to each size from top to bottom.

**Figure** **4**a presents the deformation prediction results of the CSPG algorithm across voxel encodings of different lengths. For each length category, 1000 randomly generated samples were evaluated, with each scatter point in the figure representing the MSE value of an individual sample. Overall, the MSE distributions remain relatively concentrated across different input dimensions, which indicates that the algorithm demonstrates robustness to structural scale variations. As the voxel encoding length increases, the mean MSE shows a slight upward trend. Nevertheless, the MSE values across all size categories consistently remain below 10^−5^, demonstrating that the CSPG algorithm achieves high prediction accuracy across a wide range of structural dimensions.

Figure 4b,c shows the scatter density distribution of predicted and true coordinates for 1000 samples of 2 × 30 size. Each sample contains 600 coordinate points, resulting in a total of 600 000 points used for statistical analysis. The results show a strong alignment between predicted and true values along the diagonal for both *x* and *y* coordinates. The coefficients of determination *R* ^2^ reach 0.9999, demonstrating the high accuracy of the CSPG algorithm in the deformation prediction. The scatter density plots of predicted and true coordinates for the DL methods are presented in Figure S4 (Supporting Information). Figure 4d displays the prediction results for five randomly selected samples from each length category. Each row corresponds to a specific structure length. The predicted curves almost completely overlap with the true deformation curves, visually confirming the accuracy of the CSPG algorithm across different structural scales.

### 2.4 Performance Comparison Between CSPG Algorithm and DL Methods

In this study, the voxel encoding sequences used as inputs exhibit significant sequential characteristics, while the corresponding deformation coordinates as outputs also demonstrate clear temporal continuity and order. This input–output relationship naturally forms a sequence‐to‐sequence mapping problem. For such tasks, RNNs^[^ ^51^, ^52^, ^53^ ^]^ offer inherent advantages, and their variants Long Short‐Term Memory (LSTM)^[^ ^54^, ^55^ ^]^ and Gated Recurrent Unit (GRU)^[^ ^56^, ^57^ ^]^ provide enhanced capabilities for capturing long‐range dependencies within sequences. To further evaluate the performance of the proposed CSPG algorithm, we conducted a comparative study with three representative DL models (RNN, LSTM, and GRU) to assess their effectiveness in the deformation prediction task.

For the three DL models, training and test datasets were constructed based on FE simulations. The training set contains three different sizes of encoding samples (2 × 10, 2 × 15, 2 × 20), with 1000, 3000 and 5000 samples respectively. The test set contains five sizes (2 × 5, 2 × 10, 2 × 15, 2 × 20, 2 × 30), each containing 1000 samples. All models were trained using the Adam optimizer, with the MSE employed as the loss function. The network structure and parameter design of the DL models are presented in Figure S5 (Supporting Information).

**Figure 5:**
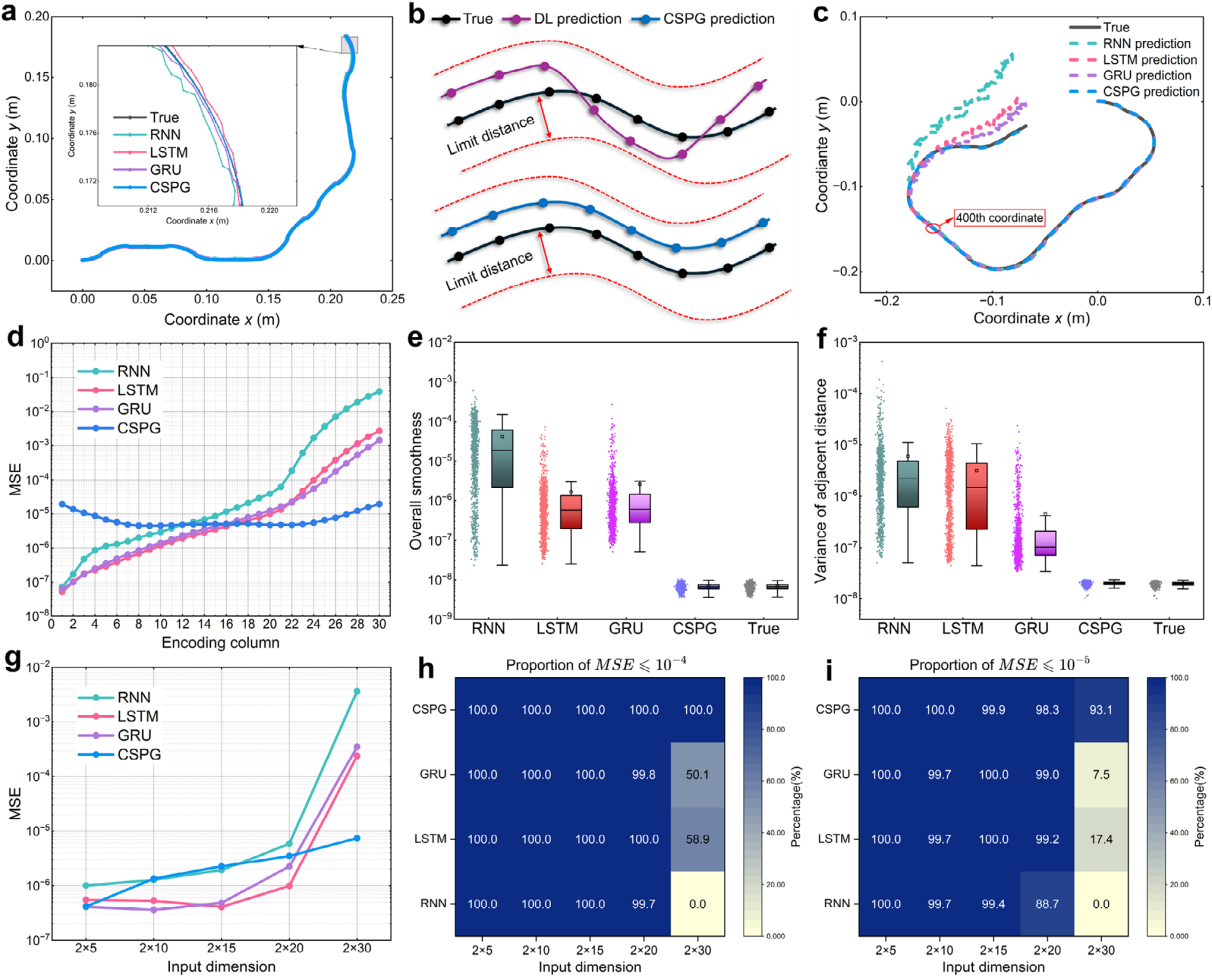
Comparison of prediction performance between the CSPG algorithm and DL methods. a) For a sample with a size of 2 × 20, the predicted trajectories generated by the three DL models are not smooth enough and deviate from the true deformation, while the trajectory predicted by the CSPG algorithm highly matches the real shape. b) Explanation for the difference in predicted trajectories: the DL method only optimizes the distance error between the coordinate points and the real points, and lacks modeling of the overall trajectory; while the CSPG algorithm relies on explicit geometric features such as curvature, enabling it to better maintain a consistent trend between the predicted and true deformation trajectories. c) For a sample with size 2 × 30 beyond the training range of the DL models, the prediction accuracy of DL methods drops significantly, while the CSPG algorithm consistently maintains high precision. d) Comparison of prediction errors across different columns of the structure for each method. The CSPG algorithm maintains consistently low errors across all columns, while DL methods exhibit significantly increased errors on columns outside the training range. e) Comparison of overall smoothness in the prediction results of each method. The CSPG algorithm produces a smoothness distribution highly consistent with the true data, whereas the results from DL methods are more scattered. f) Analysis of the variance in distances between adjacent points shows that the prediction results of the CSPG algorithm closely match the distribution of the true deformation. g) Comparison of prediction performance across different structure sizes shows that for sizes ranging from 2 × 5 to 2 × 20, the CSPG algorithm performs comparably to DL methods. However, for the 2 × 30 structures that exceed the DL training range, the CSPG algorithm demonstrates significantly higher prediction accuracy. h,i) Comparison of low‐precision and high‐precision prediction accuracy on 2 × 30 size samples shows that the CSPG algorithm significantly outperforms DL methods on both metrics.

**Figure** **5**a shows the prediction results for a sample with a voxel size of 2 × 20. At a coarse level of observation, the coordinate points predicted by the DL models and the CSPG algorithm are highly consistent with the actual deformation trajectory. However, upon magnification of the predicted curves, distinct differences become evident. Although the coordinate points predicted by the DL models are numerically close to the truth value, their predictions lack geometric smoothness and fail to accurately capture the curvature characteristics of the deformation path. In contrast, the CSPG algorithm not only achieves high accuracy but also generates geometrically smooth curves that almost completely align with the true deformation trajectory.

The differences in prediction performance can be further interpreted through Figure 5b. In such prediction tasks, DL models are typically trained using MSE as the loss function, which minimizes the Euclidean distance between predicted and true points. While this ensures numerical closeness, it overlooks geometric continuity, curvature, and inter‐point distances. As shown in Figure 5b, DL models produce points close to the truth, but fail to form smooth curves consistent with real deformation trajectories. Moreover, discrepancies in adjacent point distances result in spatial inconsistencies. In contrast, the CSPG algorithm incorporates curvature and adjacent distance features as priors during prediction, ensuring geometric smoothness and structural consistency.

Figure 5c presents the prediction results for a voxel encoding of size 2 × 30 using different methods. For the first 20 columns (points 1 ∼ 400), all three DL models and the CSPG algorithm show good alignment with the true deformation trajectory. However, in the final 10 columns (points 401 ∼ 600), the DL models display noticeable deviations, whereas the CSPG algorithm maintains high accuracy throughout. The predicted curves become severely distorted and fail to maintain a continuous and smooth structural contour. This is because the maximum voxel encoding size in the training set for the three DL models is limited to 2 × 20. When the input sequence length significantly exceeds this range, the models lack sufficient contextual understanding and generalization capability for the unseen structural segments, resulting in a substantial decline in prediction accuracy. In contrast, the CSPG algorithm incorporates curvature and inter‐point distance features to perform inference‐based prediction. This enables CSPG to generate accurate and continuous deformation coordinates for voxel encodings of arbitrary length.

To provide a more intuitive comparison of different methods in long‐sequence deformation prediction tasks, 1000 voxel‐coded samples of size 2 × 30 were input into each of the three DL models (RNN, LSTM, GRU) and the CSPG algorithm. For each sample, the predicted results were divided into 30 column‐wise segments. The MSE was computed for each column, and then averaged across 1000 samples. As shown in Figure 5d, the prediction accuracy of RNN remains below 10^−4^ for the first 21 columns. However, starting from the 22nd column, the error increases significantly, indicating a rapid decline in performance when handling longer sequences. The GRU and LSTM models perform slightly better, maintaining low error levels up to the 23rd and 24th columns, respectively, before also exhibiting marked deviations. These results suggest that, although GRU and LSTM possess a degree of long‐sequence prediction capability, they remain constrained by the memory window learned during training and exhibit limited generalization beyond that range. In contrast, although the CSPG algorithm exhibits slightly higher MSE values than the DL models in the first 10 columns, its per‐column error remains consistently below 10^−4^, generally falling within the 10^−5^ range. From the 18th column onward, CSPG begins to outperform all three DL models, with the performance gap progressively widening in the later segments. These results demonstrate that CSPG offers significant advantages in long‐sequence and even arbitrary‐length deformation prediction tasks.

For this type of deformation prediction task, although the MSE is able to quantify the numerical difference between the predicted and true coordinates, it is not sufficient to fully reflect the geometric quality of the predicted curves. Especially for the structural deformation problem, the smoothness of the prediction curve is also a critical evaluation metric. Therefore, we introduce an additional evaluation metric: curve smoothness. Specifically, the predicted curve is represented as a series of discrete coordinate points {(*x*_1_, *y*_1_), (*x*_2_, *y*_2_), …, (*x*_*n*_, *y*_*n*_)}. The local smoothness at each point is measured using the modulus $\left\|s_{i}\right\|$ of the second‐order difference vector, as defined in Equation (10). This parameter quantifies the local bending tendency of the coordinate point, where smaller values indicate less curvature and thus a smoother curve at that location.

(10) $$\begin{array}{c} \left\|s_{i}\right\|=\sqrt{{\left(x_{i+2}-2x_{i+1}+x_{i}\right)}^{2}+{\left(y_{i+2}-y_{i+1}+y_{i}\right)}^{2}} \end{array}.$$

Furthermore, by computing the variance of all local smoothness values $\left\|s_{i}\right\|$ along the curve, we obtain a global smoothness metric, denoted as $\sigma_{s}^{2}$. A smaller value of $\sigma_{s}^{2}$ indicates that the curve exhibits greater overall smoothness.

(11) $$\begin{array}{c} \sigma_{s}^{2}=Var\left(\left\|s_{i}\right\|\right) \end{array}.$$

Figure 5e presents a comparison of global smoothness between the three DL models and the CSPG algorithm. Each method was used to predict 1000 samples of size 2 × 30, with the global smoothness calculated for each sample. Each scatter point in the figure represents the smoothness value for an individual sample. The results indicate that the smoothness distributions of the curves generated by the three DL models are highly dispersed and deviate significantly from the distributions observed in the truth data. This confirms that the lack of curve smoothness observed in Figure 5a is not an isolated instance, but a common problem across the entire sample set. In contrast, the CSPG algorithm produces a concentrated smoothness distribution that closely aligns with that of the real data.

Figure 5f illustrates the comparative analysis of different methods on the variance value $\sigma_{d}^{2}$ of the distance *d*_*i*_ between adjacent coordinate points. Each method was used to predict 1000 samples of size 2 × 30. For each sample, the variance of all *d*_*i*_ values was computed to assess the uniformity of point spacing in the prediction results. The results show that the $\sigma_{d}^{2}$ distributions of the three DL models are noticeably scattered and deviate substantially from those of the truth data, indicating a lack of regularity in their predicted inter‐point distances. In contrast, the CSPG algorithm exhibits highly stable performance on this metric, with its predicted distributions closely matching those of the real data. More detailed comparisons between the CSPG algorithm and the DL methods in terms of global smoothness of the predicted results and the variance of adjacent point distances are presented in Figure S6 (Supporting Information).

Figure 5g compares the prediction performance of the CSPG algorithm with three DL models (RNN, LSTM, GRU) across voxel encodings of varying lengths. Each encoding size (2 × 5 ∼ 2 × 30) contains 1000 samples. Since the DL models were trained on data with a maximum encoding length of 2 × 20, they perform well on test sets within this range, achieving relatively high prediction accuracy. However, when the encoding length is extended to 2 × 30, the three DL models experience a sharp decline in performance, with MSE values rising above 10^−4^, reflecting their limited generalization capability beyond the training range. In contrast, the CSPG algorithm achieves prediction accuracy comparable to that of the RNN model and slightly lower than GRU and LSTM for encodings shorter than 2 × 20. However, when applied to encodings of size 2 × 30, CSPG maintains stable performance, with MSE values remaining below 10^−5^. This significantly outperforms the three DL models, further confirming the effectiveness of the algorithm in scalable deformation prediction.

To facilitate a more intuitive comparison of prediction performance under different accuracy requirements, two evaluation criteria based on the MSE are defined. Predictions with *MSE* ⩽ 10^−4^ are considered low‐precision successes, while those with *MSE* ⩽ 10^−5^ are considered high‐precision successes. Each encoding type includes 1000 samples, and the prediction success rates under these criteria are summarized in Figure 5h,i, respectively. As shown in Figure 5h, under the low‐precision criterion, the CSPG algorithm achieves a 100% prediction success rate across all five encoding lengths. The three DL models also perform well for the first four encoding sizes, with success rates approaching 100%. However, when applied to the 2 × 30 size encoding, their performance declines sharply: GRU achieves a success rate of 50.1%, LSTM reaches 58.9%, and RNN fails entirely, with a 0% success rate.

Figure 5i presents the results under the high‐precision criterion. For the three shorter encodings (2 × 5, 2 × 10, 2 × 15), both the CSPG algorithm and the three DL models achieve near 100% success rates. At the 2 × 20 length, CSPG attains a success rate of 98.3%, slightly lower than those of GRU and LSTM, but significantly higher than that of RNN. When the encoding length increases to 2 × 30, CSPG still maintains a high‐precision success rate of 93.1%, while GRU and LSTM drop sharply to 7.5% and 17.4%, respectively. RNN remains entirely ineffective, with a 0% success rate. This confirms that the CSPG algorithm demonstrates superior generalization capability, structural understanding, and predictive stability in deformation prediction tasks.

The above prediction results show that the three DL models achieve high accuracy when processing samples within the range of the training data. To further quantify the upper limit of their predictive capability regarding structural size, each model was evaluated using 1000 samples with dimensions of 2 × 30, and the MSE was calculated for each column of the predicted results. The columns with *MSE* ⩽ 10^−4^ are defined as effectively predicted columns. If the percentage of effectively predicted columns reaches more than 90%, the columns are considered to be within the effective prediction range of the model. As summarized in **Table** **1**, the amount and structural complexity of FE simulation data required for training the three DL models are significantly greater than those needed by the CSPG algorithm. This results in a total simulation time of 16 days, which is 48 times longer than that required for the CSPG algorithm. Despite this extensive data requirement, the RNN model exhibits a maximum predictable structure size of 2 × 20, which corresponds precisely to the largest structure included in its training set. This outcome indicates that the model is unable to generalize to structures beyond the scope of its training data. In contrast, the LSTM and GRU models exhibit slightly better scalability in terms of structural size, with maximum predictive limits of 2 × 23 and 2 × 24, respectively. Nevertheless, their predictive capabilities remain constrained by the scale of the training data.

**Table 1:** Comparison of the required data, simulation time, and predictable length across different methods.

Method	Required data	FE simulation time of required data	Predictable length
RNN	2 × 10 (1000)	387.5 h	2 × 1 ∼ 2 × 20
	2 × 15 (3000)		
	2 × 20 (5000)		
LSTM	2 × 10 (1000)	387.5 h	2 × 1 ∼ 2 × 23
	2 × 15 (3000)		
	2 × 20 (5000)		
GRU	2 × 10 (1000)	387.5 h	2 × 1 ∼ 2 × 24
	2 × 15 (3000)		
	2 × 20 (5000)		
CSPG	2 × 5 (640)	8 h	Arbitrary length (Theory)

Notably, the CSPG algorithm successfully predicted all structures of sizes 2 × 30 and below during testing, without requiring a large dataset. Although longer structures were not further evaluated in this study, CSPG is fundamentally an inference‐based prediction method that leverages explicit structural features. Therefore, it is theoretically capable of handling deformation prediction for voxel‐encoded structures of arbitrary length, demonstrating exceptional scalability.

In addition to prediction accuracy, the prediction speed of the three DL models and the CSPG algorithm was evaluated across different voxel encoding sizes, as summarized in **Table** **2**. For a single structure of size 2 × 5, the FE simulation requires approximately 45 seconds, whereas all DL models and the CSPG algorithm complete predictions within 0.02 ∼ 0.03 seconds per sample, demonstrating a significant computational efficiency advantage. As the structure size increases, the FE simulation time grows rapidly, reaching 135 seconds for 2 × 20, which limits its practicality for batch prediction and complex designs. In contrast, the DL models maintain consistent prediction times of less than 0.03 seconds across all sizes. Due to its progressive coordinate point generation mechanism, the CSPG algorithm exhibits prediction times that increase with structure length. When the structure size reaches 2 × 20, the single prediction time of the CSPG algorithm is about 0.17 seconds. Despite being slower than the DL models, CSPG still offers a substantial efficiency advantage over FE simulation, meeting the demands of large‐scale prediction and rapid iterative design.

**Table 2:** Time consumption (in seconds) of different methods on various structure sizes.

Method	Time consumption			
	2 × 5	2 × 10	2 × 15	2 × 20
FE	45s	86s	110s	135s
RNN	0.0235s	0.0235s	0.0242s	0.0253s
LSTM	0.0255s	0.0252s	0.0258s	0.0261s
GRU	0.0225s	0.0247s	0.0253s	0.0270s
CSPG	0.0263s	0.0732s	0.1149s	0.1664s

### 2.5 Deformation Prediction of 4D Printed Hydrogels Using CSPG Algorithm

The deformation prediction method proposed in this study is based on an intelligent hydrogel capable of forming voxel structures. This hydrogel exhibits solvent‐responsive behavior, undergoing significant swelling when immersed in water. Using 4D printing technology, the swelling ratio of each voxel unit can be controlled. In this experiment, voxel units encoded as 1 exhibited a linear swelling ratio of 2.1223, while those encoded as 0 demonstrated a swelling ratio of 1.8938. Due to these differences in swelling behavior among the encoded units, the hydrogel undergoes pronounced shape transformations during the swelling process. Details of the chemical composition and preparation methods of the hydrogel are provided in Figure S7 and Table S1 (Supporting Information). Details of the printing equipment and printing parameters are presented in Figure S8 and Table S2 (Supporting Information). Figures S9 and S10 (Supporting Information) illustrate the printing process.

**Figure 6:**
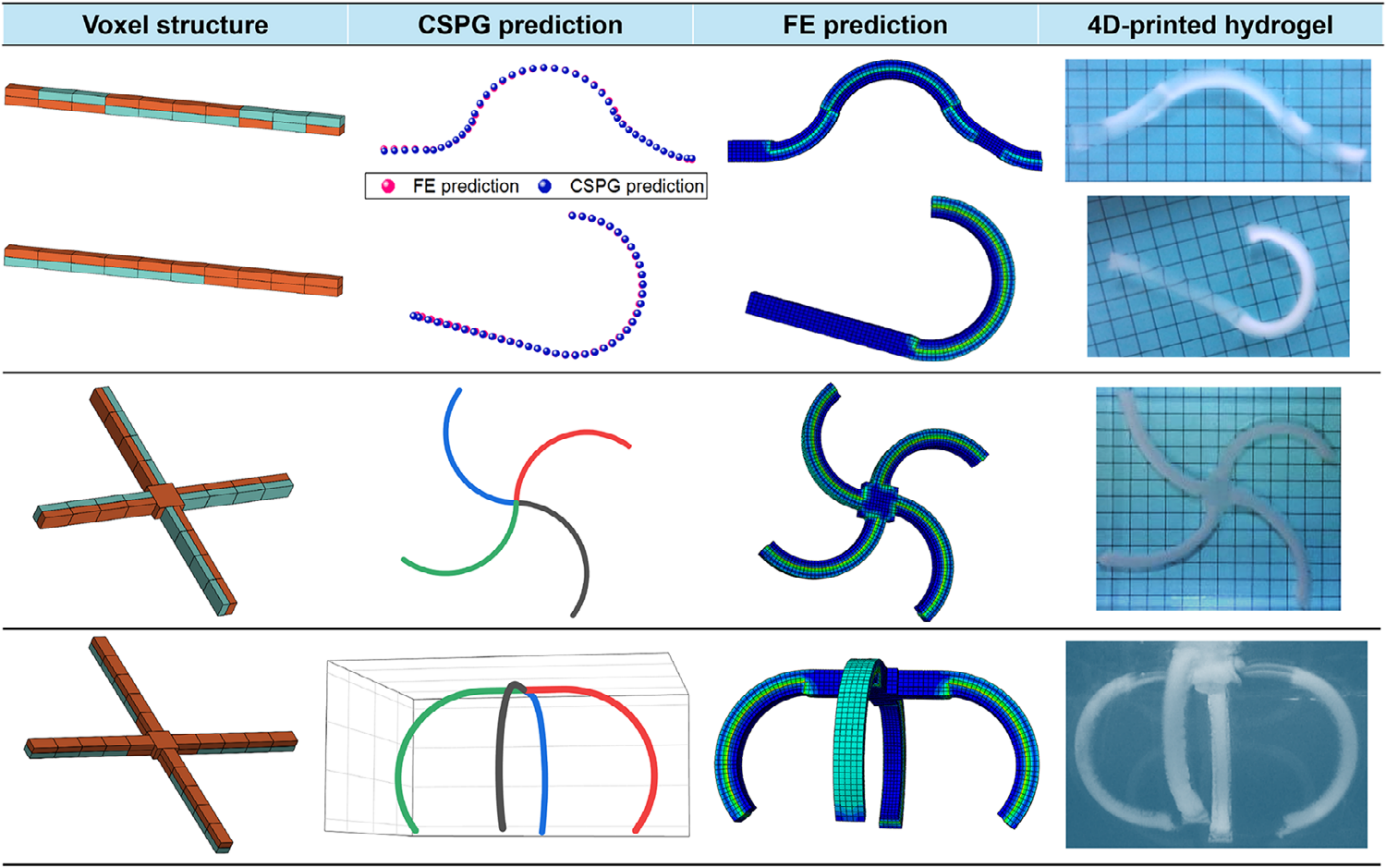
Deformation prediction of 4D‐printed hydrogel using the CSPG algorithm. A smart hydrogel capable of forming voxel structures is used as a carrier, and the prediction consistency is evaluated by comparing the deformation results predicted by the CSPG algorithm with the actual deformation of the hydrogel. The figure shows a comparison of deformation predictions for four voxel structures, presenting the CSPG algorithm prediction results, the FE simulation results, and the corresponding experimental observations.

To verify the accuracy and reliability of the CSPG algorithm in deformation prediction, four different voxel structures were designed, and the corresponding hydrogel samples were fabricated by 4D printing, as shown in **Figure** **6**. Among them, the first two voxel structures are 2 × 10 beam structures. The experimental results show that the deformation predicted by the CSPG algorithm is in perfect agreement with the FE simulation results. The shape of the hydrogel after swelling is also highly consistent with the predicted shape. In addition to 1D beam structures, we also assembled multiple beams to form 2D and 3D structures. In the FE simulations, the connection points of the 2D and 3D structures were modeled as solid blocks with dimensions of 9mm × 9mm × 5mm to ensure stress continuity and connection stability between adjacent beam elements. The material property of each connection block was encoded as “1,” meaning that the blocks underwent swelling but did not bend during the simulations. When using the CSPG algorithm for prediction, each connection point was treated as a common origin. The voxel sequence of each beam was generated outward from this origin according to predefined angles and directions. The deformation curve of each beam was independently computed by the CSPG algorithm, and all beams were subsequently assembled in 3D space via the common origin to form the complete 3D structure.

As shown in Figure 6, the third structure consists of four 2 × 4 beams spliced together. After water absorption and swelling, it forms a windmill‐like shape. The CSPG prediction in this case is consistent with the FE simulation results and is generally in agreement with the actual deformation. The fourth voxel structure consists of four 2 × 5 beam structures spliced together. Unlike the third structure, its orientation was rotated during printing so that it deforms along the z‐axis direction, eventually forming a shape similar to a 3D gripper after swelling. The results indicate that the deformation predicted by the CSPG algorithm remains highly consistent with the FE simulation results, and the actual deformation of the hydrogel closely matches the predicted results.

The above results demonstrate that the CSPG algorithm can produce deformation predictions that are highly consistent with FE simulations for both beam and spliced structures. For the actual hydrogel deformation, due to the limited accuracy of the printing equipment, the expansion rate of the fabricated voxel units deviated from the designed target, resulting in localized discrepancies between the actual deformation and the predicted results. However, these differences do not affect the practical applicability and effectiveness of the proposed method. We believe that with sufficiently high‐precision printing equipment, the deformation predictions generated by the CSPG algorithm will achieve an even closer agreement with experimental observations.

As illustrated in Figure 6, the CSPG algorithm is not limited to deformation prediction of single beam‐like structures; it is equally capable of efficiently and accurately predicting deformations in diverse voxel configurations composed of beam elements with arbitrary lengths and numbers. To further improve the practicality of this approach, an interactive web‐based prediction platform was developed. This platform enables users to define voxel encodings and assembly schemes through an intuitive visual interface and perform real‐time deformation predictions using the CSPG algorithm. For a detailed description of the prediction platform, refer to Figure S9 (Supporting Information).

## 3 Discussion

In this study, we propose a deformation prediction method for 4D‐printed active composite structures based on data mining. The method involves manually extracting geometric features that capture the relationship between 2D voxel encodings and deformation coordinates, which are subsequently used to construct a comprehensive feature database. Building upon this, a predefined sequential advancement strategy is employed to invoke the relevant feature information, and the proposed CSPG algorithm is utilized to progressively generate the complete deformation trajectory. Compared to traditional FE methods, the CSPG algorithm eliminates the need for complex physical modeling and numerical iteration, enabling each prediction task to be completed within one second and significantly enhancing prediction efficiency. In contrast to DL methods, CSPG relies solely on geometric features extracted from a limited number of samples to achieve efficient deformation prediction for voxel‐encoded structures of arbitrary lengths, thereby overcoming the limitations of DL models in terms of structure size scalability. In particular, CSPG significantly outperforms DL models in terms of prediction accuracy for long structural sequences, and also demonstrates comparable or even superior performance for short sequences.

To further enhance the practicality of the proposed method, an interactive web‐based prediction platform was developed, allowing users to customize input voxel encodings through a visual interface. This platform enables structural deformation prediction by invoking the CSPG model, thereby establishing an end‐to‐end, visualized prediction workflow. This work not only provides an efficient tool for the deformation prediction of 4D‐printed structures, but also introduces a novel approach for the optimal design and programmable manufacturing of complex smart structures.

However, for more complex 2D, 3D, or irregular structures, the CSPG algorithm still has limitations and cannot yet directly perform modeling or prediction. In future work, we plan to incorporate both bending and twisting in 3D space, as well as deformations occurring in multiple directions, enabling the algorithm to capture surface and volumetric deformations. At the same time, we will maintain its training‐free and geometry‐driven characteristics, extending it from 1D beam components to more complex 2D and 3D structures. This advancement will further support its practical application in 4D printing scenarios, such as intelligent manufacturing and functional material development.

## 4 Experimental Section

### Generation of Datasets by FE Simulation

The dataset used in this study was generated through FE simulations using ABAQUS (version 2023). The entire modeling and simulation process was automated via custom Python scripts executed within the ABAQUS environment. The target structure is a beam‐shaped composite with a fixed width and thickness of 5 mm, while the length varies according to experimental requirements, specifically 50, 100, 150, 200, and 300 mm. These correspond to voxel encoding dimensions of 2 × 5, 2 × 10, 2 × 15, 2 × 20, and 2 × 30, respectively. Each voxel unit has a physical size of 10mm × 5mm × 2.5mm. Figure S12 (Supporting Information) illustrates the meshing of the voxel structures. Figure S13 (Supporting Information) shows the boundary condition settings during FE simulation. All voxel encodings were randomly generated using Python to ensure diversity and randomness across the dataset.

In the FE simulation, thermal expansion was employed to approximate the swelling behavior of hydrogel materials upon water absorption. Regions encoded as “1” represent active materials, assigned a thermal expansion coefficient of 0.021223, while regions encoded as “0” correspond to passive materials, with a coefficient of 0.018938. To simulate the swelling process, a thermal load was applied by gradually increasing the temperature from 0°*C* to 100°*C*, with each increment limited to a maximum of 15°*C*. Under these conditions, the active material exhibits a volumetric expansion ratio of approximately 2.1223, whereas the passive material expands by a factor of 1.8938. This differential expansion effectively replicates the deformation characteristics induced by water absorption in hydrogel structures.

During the simulation, boundary conditions were applied to fix the left end of the hydrogel structure, ensuring positional stability throughout the loading process. After the simulation was completed, nodes were uniformly sampled along the deformed outer surface of the structure to represent its deformation trajectory. These voxel encodings, along with their corresponding coordinate data, form the dataset used in this study. This dataset supports both feature extraction and deformation prediction tasks for the CSPG algorithm, as well as the training and testing of DL models.

### Rigid Alignment of Predicted Shapes

In this study, the extracted geometric features are constructed based on the average features of multiple samples, aiming to capture the global morphological features of the target structure. However, the averaged features cannot accurately represent the specific geometric morphology of individual samples, which may lead to some angular deviation between the predicted shape and the real shape during the prediction process. Considering that this study mainly focuses on the consistency between the predicted results and the real results in shape rather than the absolute spatial location correspondence, we introduce a rigid transformation when evaluating the prediction accuracy. This allows the predicted and true shapes to be optimally aligned, enabling a more essential comparison of shape similarity. Specifically, let the 2D coordinate point set of the real shape be ***A*** and the point set of the predicted shape be ***B***. We expect the point set ***B*** to align with ***A*** through rotation and translation, i.e., to satisfy Equation (12):

(12) $$\begin{array}{c} A\approx R\cdot B+t \end{array},$$

where ***R*** denotes the rotation matrix and ***t*** represents the translation vector. This transformation is obtained by performing Singular Value Decomposition (SVD) on the covariance matrix between the decentered point sets. Finally, the MSE between the transformed point set $B^{\prime }=R\cdot B+t$ and set ***A*** is computed as the evaluation metric. For DL methods, the modeling mechanism is based on an end‐to‐end data‐driven learning process, where the mapping between inputs and outputs is directly learned from a large number of samples during training. In this process, the spatial pose information of each training sample is preserved and utilized by the model, allowing the predicted shapes to generally maintain angular consistency with the ground‐truth shapes. Therefore, applying rigid registration to the DL predictions is not only unnecessary but may also introduce additional errors due to the extra rotation and translation operations.

## Author Contributions

M.W. contributed to the original draft, conceptualization, visualization, methodology, and data curation. Y.X. participated in data curation, validation, and visualization. Z.L. contributed to validation and visualization. H.F. was involved in data curation and validation. Z.W. contributed to writing—review and editing, and provided supervision, resources, and funding acquisition. R.X. contributed to writing—review and editing and supervision. L.M. contributed to writing—review and editing, supervision, resources, and funding acquisition.

## Conflict of Interest

The authors declare no conflict of interest.

## Supporting information



Supporting Information

Supplemental Movie 1

Supplemental Movie 2

## Data Availability

The data that support the findings of this study are available from the corresponding author upon reasonable request.
