# Supplementary material for: Deformation Prediction of 4D‐Printed Active Composite Structures Based on Data Mining
Source: Adv Sci (Weinh). 2025 Nov 26;13(7):e16989. doi: 10.1002/advs.202516989 (PMC12866864; doi:10.1002/advs.202516989)
Supplement: Supplementary file 1 — Supporting Information [file ADVS-13-e16989-s001.pdf]

Supporting Information

Deformation Prediction of 4D-Printed Active Composite Structures Based on Data Mining

Mengtao Wang, Yifan Xu, Zaiyang Liu, Hidemitsu Furukawa, Zhongkui Wang,* Ren Xu,* and Lin Meng*

This PDF file includes:
Figure S1 to S15, Table S1 to S2

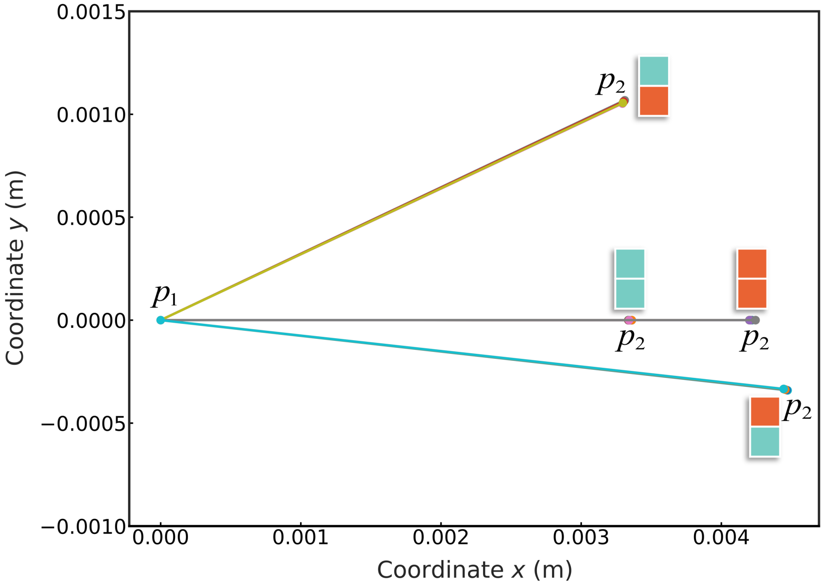

Figure S1. **Illustration of the initial two coordinate points (Feature 1).** The initial two coordinate points are extracted from a deformation dataset comprising 640 samples, each with a size of 2×5. The position of p_1 is fixed across all samples. Due to the four possible encoding combinations in the first column, the corresponding position of p_2 exhibits four distinct spatial variations.

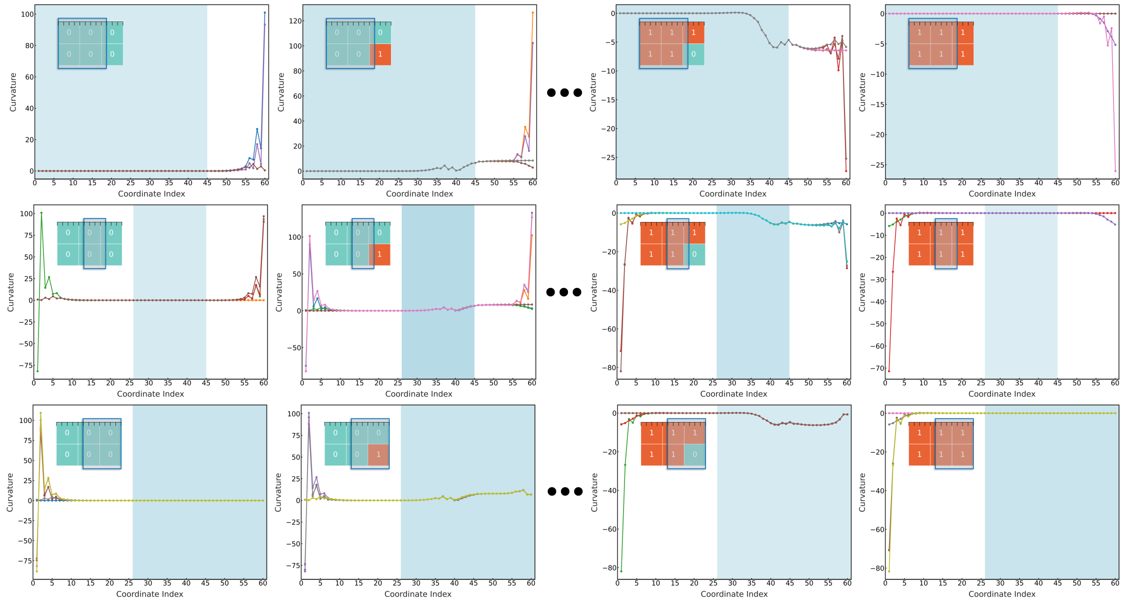

Figure S2. **Illustration of curvature feature extraction from different encoded segments.** The figure shows the curvature feature extraction process for three encoded segments: the first row corresponds to the first three columns of the encoding, the second row to the middle three columns, and the third row to the last three columns. The blue-shaded region represents the extracted feature domain. As observed, feature values across different samples in this region are highly consistent, demonstrating strong stability. The final extracted feature is obtained by averaging the values within this region across all samples. This characteristic provides a solid foundation for the construction of the CSPG algorithm and its high-precision prediction performance.

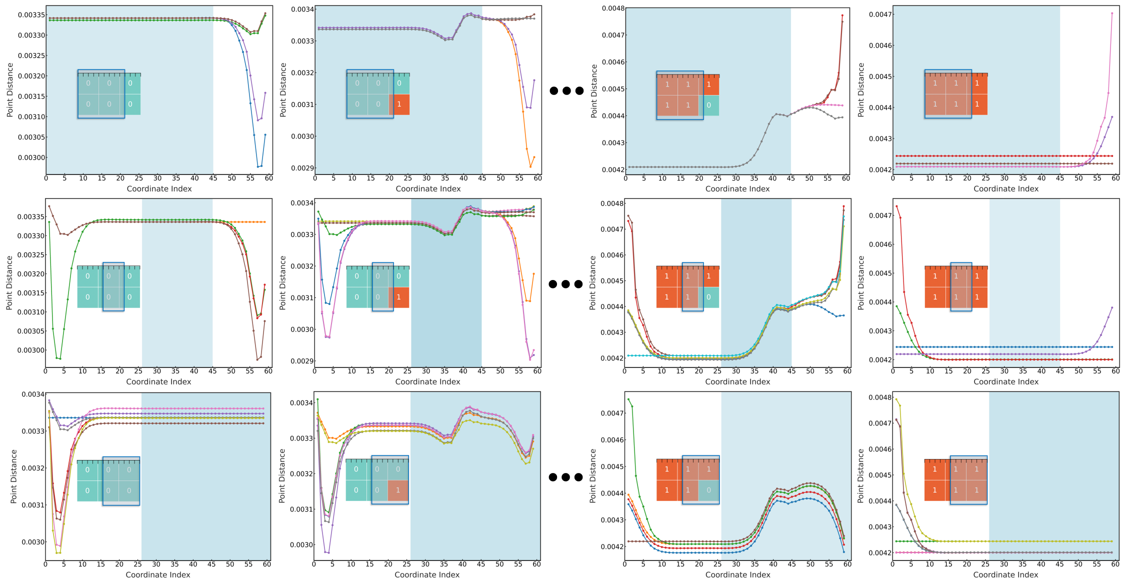

Figure S3. **Illustration of point-to-point distance feature extraction from different encoded segments.** The figure illustrates the extraction of distance features from three encoding segments: the first row corresponds to the first three columns of the encoding, the second row to the middle three columns, and the third row to the last three columns. The blue-shaded region indicates the selected feature domain. Although the feature values across different samples are not perfectly aligned within this region, their numerical deviations are small and the overall variation trends remain consistent. The final extracted feature is computed as the average of the values in this region across all samples. This characteristic not only supports the construction of the CSPG algorithm and its high prediction accuracy, but also explains the primary source of prediction error.

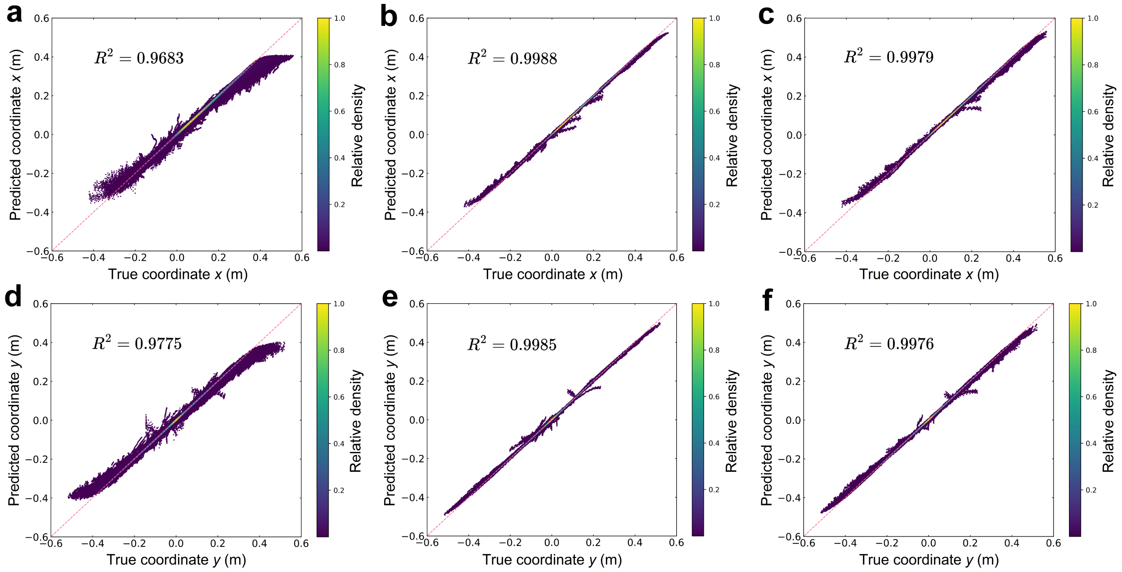

Figure S4. **Scatter density plots of predicted and true coordinates for three DL models.** Using 1,000 structural samples with a size of 2×30, scatter density plots are generated to compare the predicted coordinates with the ground truth for three deep learning models. The first, second, and third columns correspond to the RNN, GRU, and LSTM models, respectively. All three models exhibit varying degrees of deviation from the true coordinates. A comparative analysis of prediction accuracy indicates the performance ranking: RNN $<$ LSTM $<$ GRU.

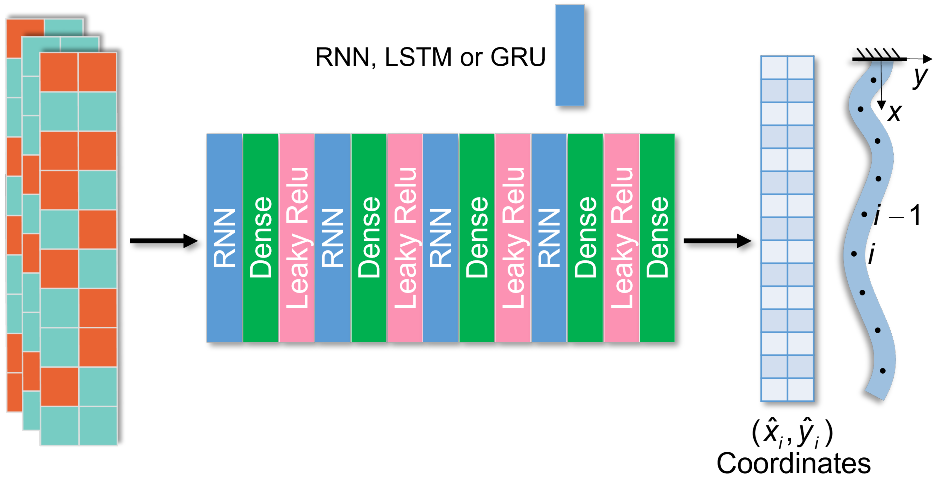

Figure S5. **Network structure of three DL models (RNN, LSTM, GRU).** The models are optimized using the Adam optimizer with a fixed learning rate of 10^{-4} and a batch size of 60.

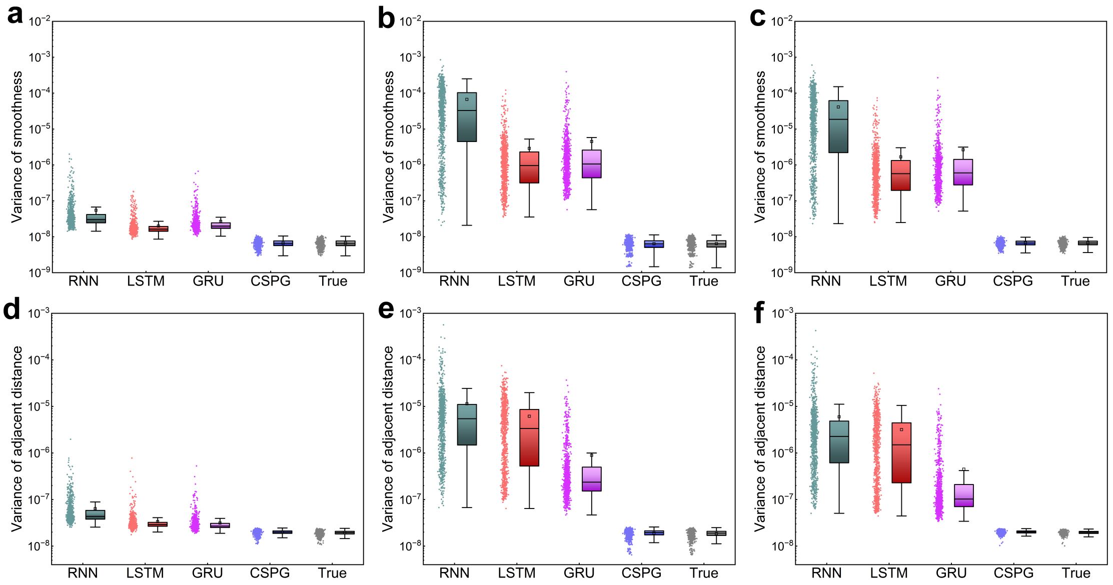

Figure S6. **Comparison of prediction smoothness and point distance variance across different encoding segments.** Using 1,000 voxel structure samples of size 2×30 as the test set, the prediction performance of three deep learning models and the CSPG algorithm is evaluated across different encoding segments. Each column corresponds to the first 20 columns, the last 10 columns, and the complete encoding, respectively. **a,b,c** The prediction smoothness comparison indicates that the three deep learning models exhibit slight deviations in the early segment and significant errors in the later and full segments. In contrast, the CSPG algorithm maintains high consistency with the ground truth across all segments. **d,e,f** The point distance variance results show a similar trend, further validating the superiority of the CSPG algorithm in prediction stability.

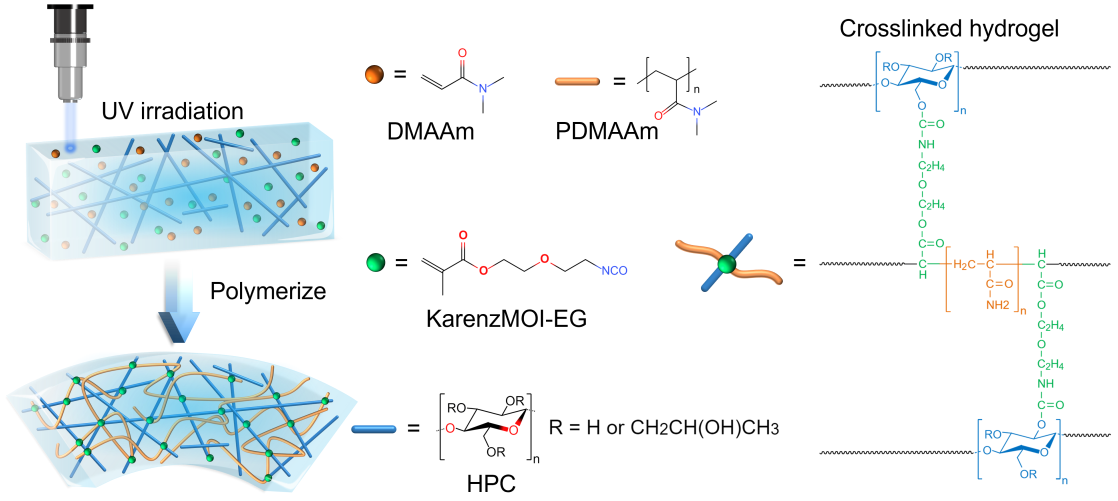

Figure S7. **Schematic of hydrogel 4D printing.** Gel solutions consisting of various compounds polymerize into crosslinked hydrogels by self-reaction after UV irradiation. Hydrogel exhibits a higher crosslink density on the side exposed to UV light and a lower crosslink density on the other side. Two sides of the hydrogel exhibit different expansion rates, and thus, it changes shape after expanding by absorbing water.

Table S1. Hydrogel materials formulation. To ensure structural stability during printing and stimulus response, the hydrogel solution is prepared strictly in accordance with the specified formulation and preparation procedures. The material dosages are accurately controlled to five decimal places to ensure consistent material properties across batches.

Number	Material Name	Dosage(g)
1	DMAAm	396.52
2	HPC	3.6032
3	KarenzMOI-EG	3.984
4	TPO	5.2257
5	Purified water	578.40
6	Absorber	1.97547

In this study, a hydrogel with solvent-responsive properties is used as a carrier to validate the effectiveness of the proposed CSPG algorithm in the deformation prediction of voxelized composite structures. This hydrogel can be used to achieve precise control of the voxelized structure using the 4D printing technique. Figure S7 shows the chemical composition of the hydrogel and a schematic diagram of the 4D printing process. The main components of the hydrogel include the hydrophilic monomer DMAAm (N,N-dimethylacrylamide), the cross-linking agent KarenzMOI-EG (2-(2-methacryloyloxyethyl)ethyl isocyanate), and the polymer HPC (hydroxypropyl cellulose). In addition, the formulation contains the hydrophobic initiator TPO (diphenyl (2,4,6-trimethylbenzoyl) phosphine oxide) to initiate the polymerization reaction, the hydrophilic initiator TPO (lithium phenyl (2,4,6-trimethylbenzoyl) phosphite), and the ultraviolet (UV) absorber AS150 (KAYAPHOR AS150).

The raw material sources were as follows: DMAAm was purchased from Tokyo Kasei Kogyo Co (Tokyo, Japan); HPC was supplied by Wako Pure Chemical Industries, Ltd (Osaka, Japan); KarenzMOI-EG was purchased from Showa Denko K.K (Tokyo, Japan); both TPO initiators were purchased from Tokyo Kasei; the UV absorber AS150 was supplied by Nippon Kayaku Co (Tokyo, Japan). The 4D printing equipment used in this study was supplied by Yuetsuki Co (Yamagata, Japan).

During hydrogel preparation, the HPC powder was first dissolved in the DMAAm solution and stirred continuously for 24 hours to ensure complete dissolution. The crosslinker KarenzMOI-EG was then added, followed by an additional 30 minutes of stirring to promote the reaction. Subsequently, purified water was introduced to terminate the reaction. The two TPO initiators and the UV absorber AS150 were then added to the mixture and stirred for another 30 minutes to complete the preparation of the hydrogel precursor solution. To ensure structural stability during both the printing process and the stimulus response phase, the entire preparation procedure strictly followed a standardized formulation. All components were measured with a precision of five decimal places to ensure consistent material properties across different batches. The detailed formulation is provided in Table S1.

Table S2. UV light parameters used for 4D printing.

Parameter of UV light	Value
Wavelength	405nm
Scanning speed	50mm/s
Spot diameter	0.3mm
Intensity	10mW

During the printing process, structural design was accomplished by precisely regulating the swelling behavior of each voxel unit. In particular, voxel units encoded as “1” were subjected to three UV exposures to achieve a higher swelling rate, whereas those encoded as “0” received only a single scan to retain a lower swelling rate. The detailed UV light parameters are presented in Table S2. Upon completion of printing, the hydrogel samples were immersed in purified water and tested for swelling behavior under standard atmospheric pressure at a room temperature of $25°C$. The resulting deformation was monitored to evaluate consistency with the FE simulation predictions.

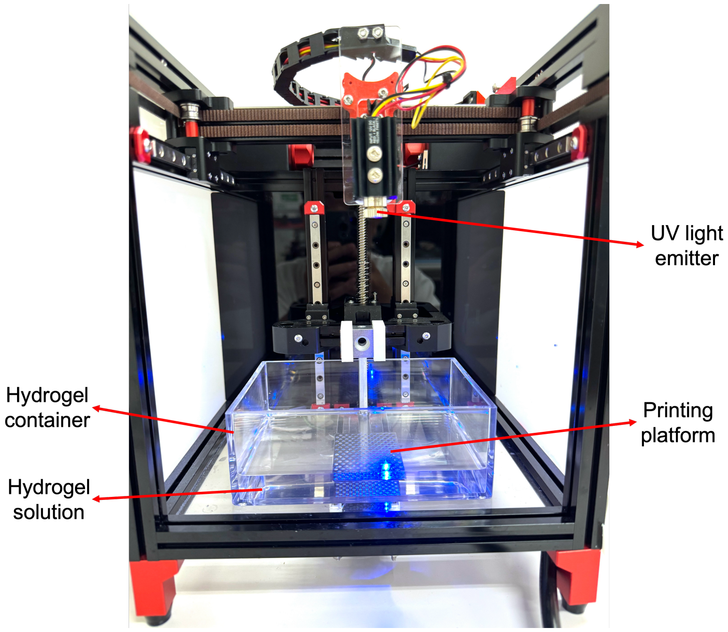

Figure S8. **4D printing equipment.** The 4D printing equipment is a first-generation printer developed by the team of Professor Hidemitsu Furukawa at Yamagata University. It comprises a UV light source, a reservoir for hydrogel, a movable printing platform, and the hydrogel solution. The UV light emitter is capable of moving along the x and y axes to scan and cure the hydrogel, while the printing platform adjusts vertically along the z-axis to build the structure layer by layer.

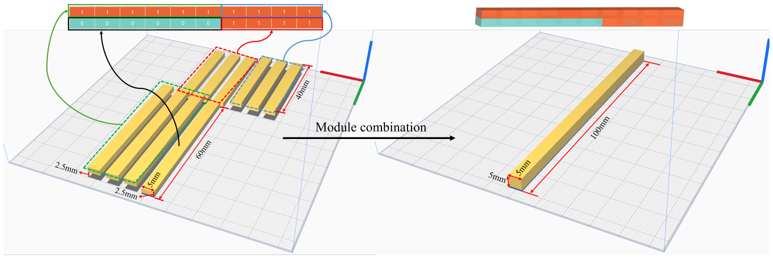

Figure S9. **Model construction of 4D-printed hydrogels.**

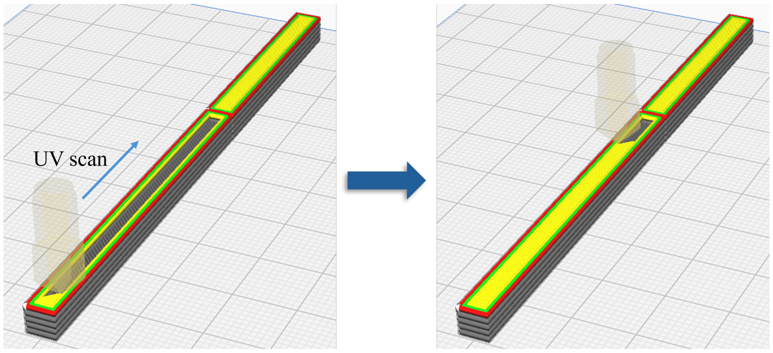

Figure S10. **UV light scanning diagram.**

During the 4D printing process of hydrogels, the hydrogel solution undergoes a light-initiated polymerization reaction under UV irradiation, forming a crosslinked hydrogel structure. In this study, we kept the UV light scanning intensity and speed constant while controlling the material properties of each voxel unit by changing the number of scans. As shown in Figure S9, in the 3D printing software (UltiMaker Cura), we represented the number of UV light scans by stacking yellow blocks. The encoding "1" represents three UV light scans, indicated by stacking three yellow blocks, while the encoding "0" represents one scan, indicated by one yellow block. By stacking these modules, we constructed a beam-shaped structure with dimensions of 100mm in length, 5mm in width, and 5mm in height.

Figure S10 shows the UV light scanning method. First, UV light follows the contours of the model (red and green areas) to print the outer contours, and then the yellow area is scanned layer by layer to complete the overall printing. This printing method can precisely control the material properties of each voxel unit and ensure the uniformity of the material distribution.

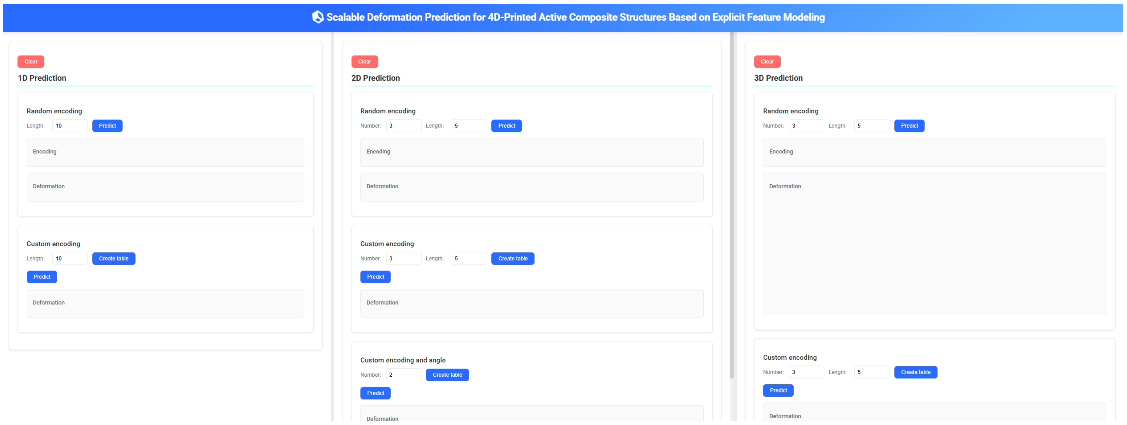

Figure S11. **Interactive web-based prediction platform based on the CSPG algorithm.** According to the type of deformation, the platform provides one-dimensional (1D), two-dimensional (2D), and three-dimensional (3D) prediction capabilities. 1D deformation prediction refers to the prediction of a single beam-like structure, with the results presented as a one-dimensional curve, hence designated as 1D prediction. 2D deformation prediction is applied to planar structures composed of multiple assembled beam elements without significant deformation along the z-axis, with the results represented as a two-dimensional plane formed by multiple 1D curves. 3D deformation prediction is similarly based on assembled beam elements but includes deformation along the z-axis, resulting in three-dimensional structures composed of multiple one-dimensional curves. The platform supports customization of beam length and encoding for 1D predictions. For 2D and 3D predictions, users can flexibly define the number of assembled beam elements, the encoding of each beam, and the angles between adjacent beams, enabling personalized deformation prediction and visualization. Supplementary Movie 2 demonstrates the use of this prediction platform.

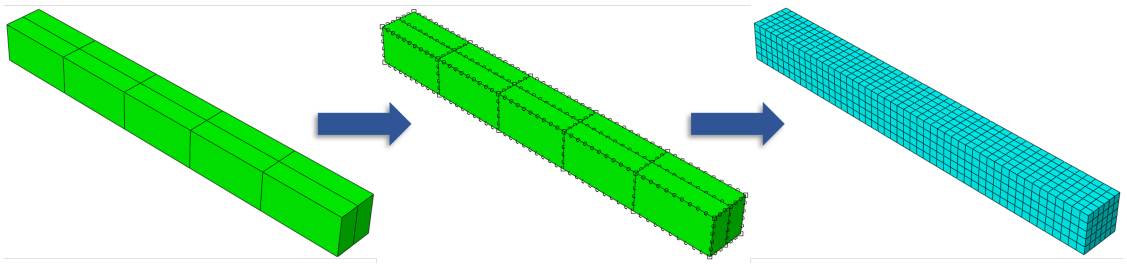

Figure S12. **Meshing of voxel structures.**

In the FE simulation, the commercial software Abaqus was used to perform the analysis, and the meshing scheme is illustrated in Figure S12. The figure depicts a 2×5 voxel structure, where the global element size was set to 1 mm and the minimum element size to 0.1 mm. Each voxel was automatically divided into $10 \times 5 \times 3$ uniform hexahedral elements, with individual element dimensions of $1\text{mm} \times 1\text{mm} \times (5/6)\,\text{mm}$. Under this meshing scheme, the simulation results closely matched the experimental results, accurately capturing the actual deformation of the printed structure.

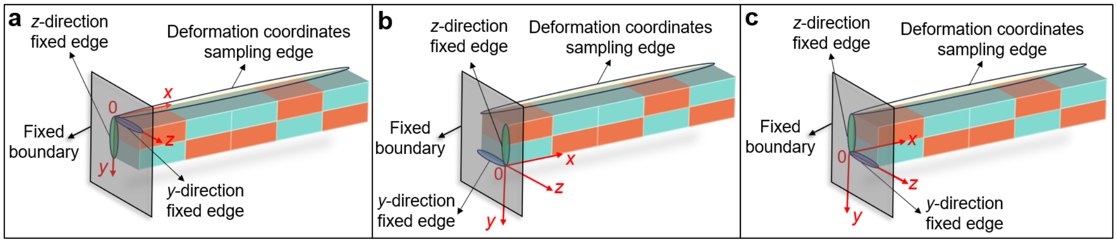

Figure S13. Different boundary condition settings in FE simulation.

Figure S13a illustrates the boundary condition settings in the FE simulation. The left end of the voxel beam is connected to a fixed boundary, where the contacting surface has no displacement in the x-direction and cannot rotate (U1 = UR2 = UR3 = 0). The green-marked edge has no displacement and no rotation in the z-direction (U3 = UR1 = UR2 = 0), while the blue-marked edge has no displacement and no rotation in the y-direction (U2 = UR1 = UR3 = 0). The yellow-marked edge is defined as the deformation coordinate sampling edge, where 20 coordinate points are extracted along each voxel to describe the deformation profile. All data and examples in this paper are based on the boundary conditions shown in Figure S13a. This configuration ensures that the deformation coordinate points of all structures reside within the same coordinate system, facilitating feature extraction and deformation prediction.

The proposed CSPG algorithm is equally applicable under different boundary conditions, but some conditions must be satisfied. First, the boundary conditions must not affect the overall deformation behavior of the structure. Second, the FE simulation data used for feature extraction must be maintained under the same boundary conditions. Although different boundary conditions do not alter the values of curvature k and point distance d, they do affect the positions of the initial coordinate points p_1 and p_2 corresponding to the first column of the encoding. The CSPG algorithm precisely relies on p_1, p_2, and their corresponding k and d values to recursively compute all deformed coordinate points $p_3, p_4, \cdots$.

For deformation prediction, the CSPG algorithm can still achieve accurate predictions even when test data employs different boundary conditions. As shown in Figures S13b and S13c, different boundary conditions were applied compared to Figure S13a, while the deformation coordinate sampling edges remained consistent. For the case in Figure S13b, we randomly generated two sets of voxel encodings and performed FE simulations and CSPG predictions. Figure S14 shows the results, where a positional offset is observed between the FE and CSPG curves due to boundary differences; however, after rigid alignment (rotation and translation), the two curves nearly coincide. For Figure S13c, two voxel encodings were generated, and the prediction results are presented in Figure S15. Similarly, after alignment, the CSPG and FE results almost perfectly overlap. Therefore, for other types of boundary conditions, as long as they do not alter the overall deformation behavior, the CSPG algorithm can still achieve precise deformation prediction.

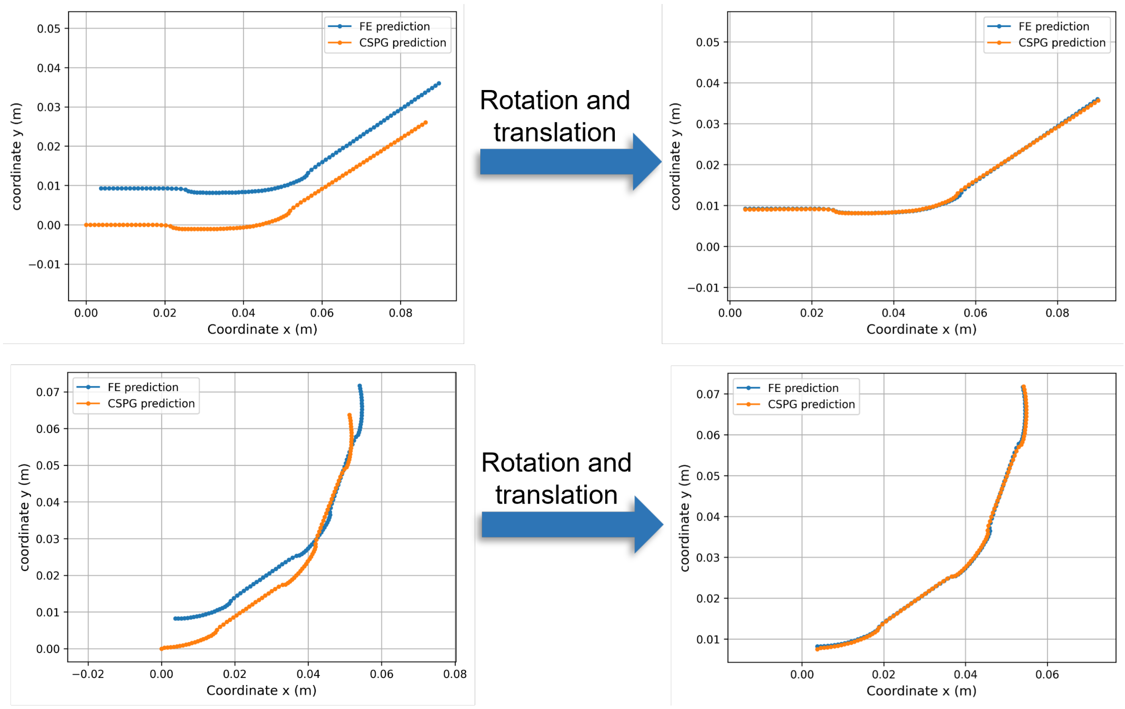

Figure S14. Prediction results under the boundary condition shown in Figure S13b.

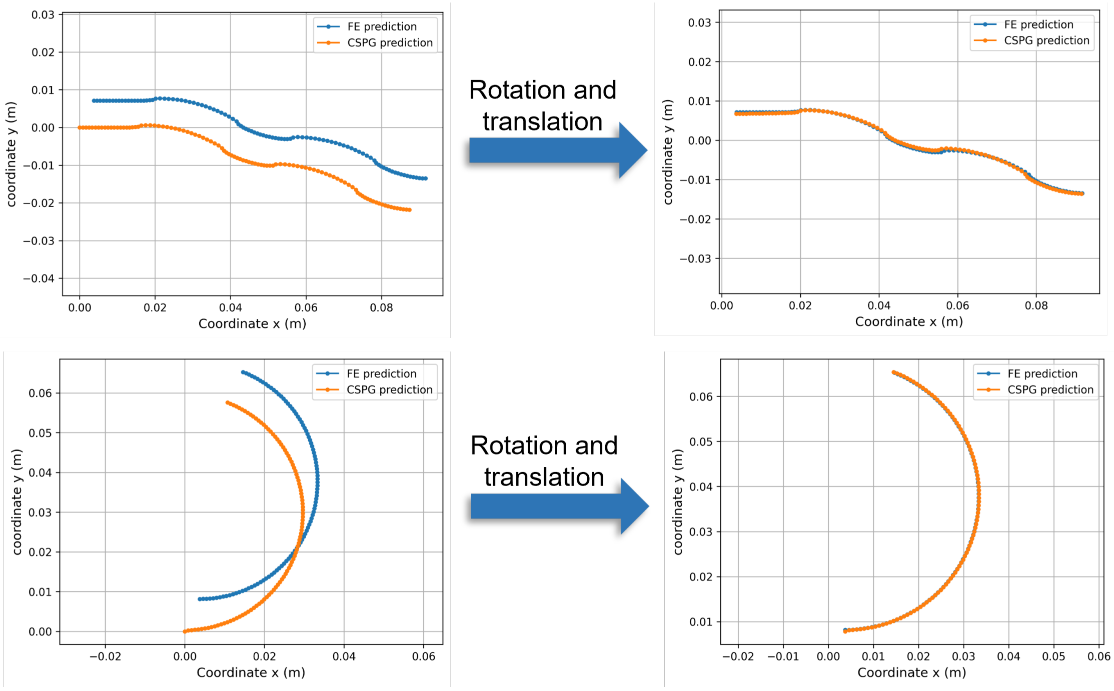

Figure S15. Prediction results under the boundary condition shown in Figure S13c.
